# Proteolysis-targeting chimaeras (PROTACs) as pharmacological tools and therapeutic agents: advances and future challenges

**DOI:** 10.1080/14756366.2022.2076675

**Published:** 2022-06-14

**Authors:** Chao Wang, Yujing Zhang, Tingting Zhang, Lingyu Shi, Zhongmin Geng, Dongming Xing

**Affiliations:** a The Affiliated Hospital of Qingdao University, Qingdao University, Qingdao Cancer Institute, Qingdao, China; b The Affiliated Cardiovascular Hospital of Qingdao University, Qingdao University, Qingdao, China; c School of Pharmacy, Qingdao University, Qingdao, China; d School of Life Sciences, Tsinghua University, Beijing, China

**Keywords:** PROTACs, targeted protein degradation, in clinical, promising treatment

## Abstract

Proteolysis-targeting chimaeras (PROTACs) have been developed to be an emerging technology for targeted protein degradation and attracted the favour of academic institutions, large pharmaceutical enterprises, and biotechnology companies. The mechanism is based on the inhibition of protein function by hijacking a ubiquitin E3 ligase for protein degradation. The heterobifunctional PROTACs contain a ligand for recruiting an E3 ligase, a linker, and another ligand to bind with the protein targeted for degradation. To date, PROTACs targeting ∼70 proteins, many of which are clinically validated drug targets, have been successfully developed with several in clinical trials for diseases therapy. In this review, the recent advances in PROTACs against clinically validated drug targets are summarised and the chemical structure, cellular and *in vivo* activity, pharmacokinetics, and pharmacodynamics of these PROTACs are highlighted. In addition, the potential advantages, challenges, and prospects of PROTACs technology in disease treatment are discussed.

## 1. Introduction

**Figure 1.**
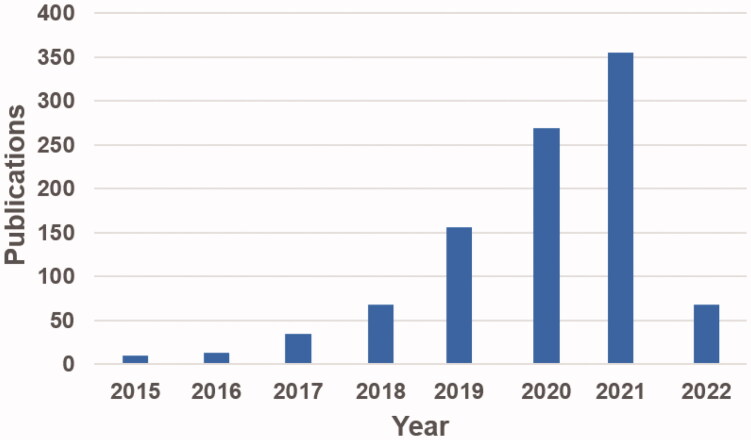
The number of publications on PROTACs in PubMed (accessed on 21 February 2022).

**Figure 2.**
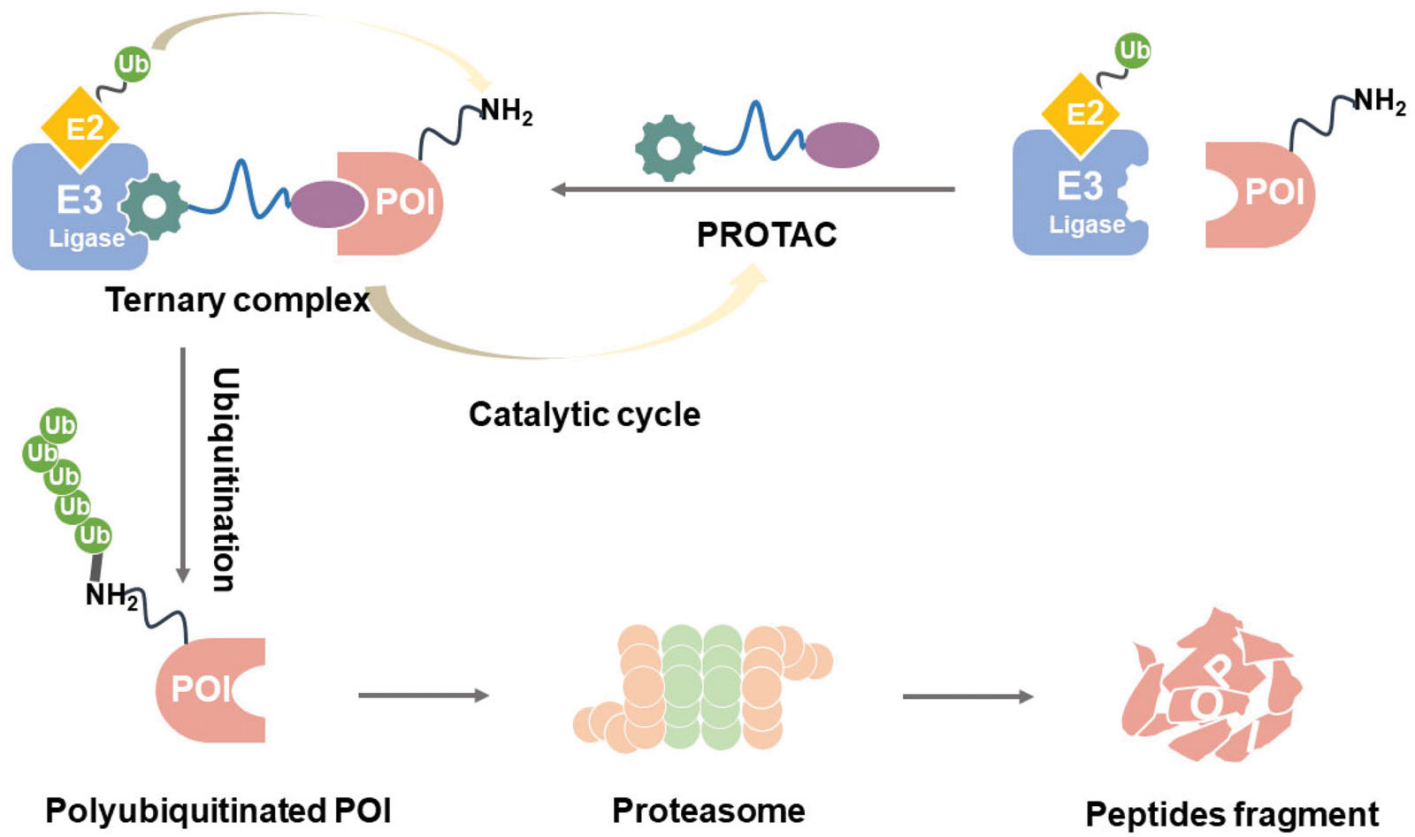
The schematic diagram of PROTACs.

**Figure 3.**
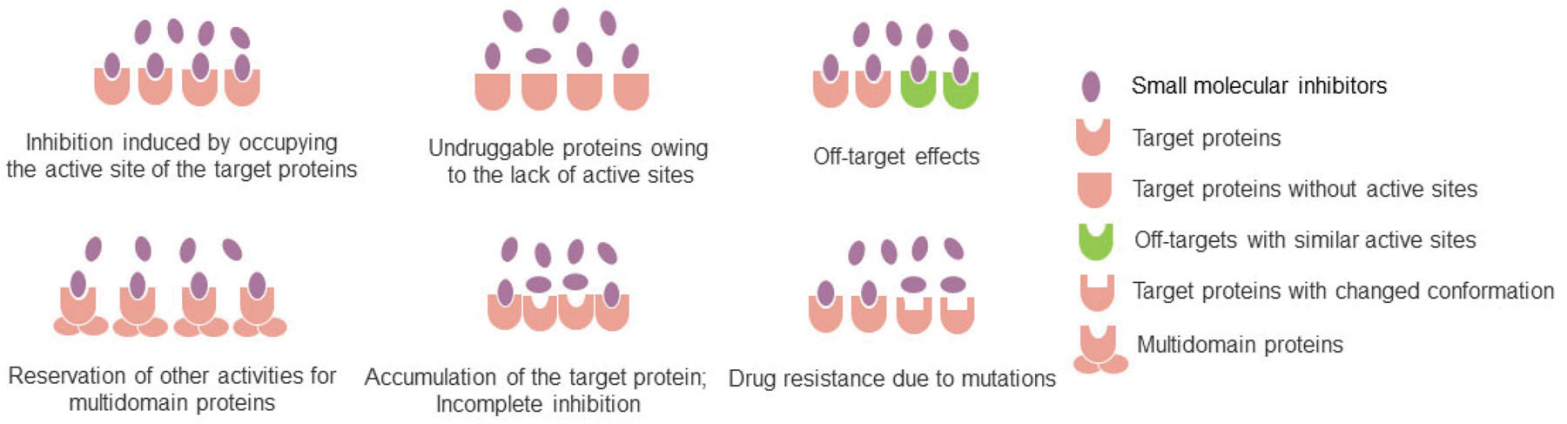
The limitations of small-molecule inhibitors.

Proteolysis-targeting chimaeras (PROTACs) are a new technique for chemical knockdown of proteins of interest (POI) that have attracted increasing research interest in recent years (Figure 1).^1–4^ PROTACs consist of three specific elements: an E3 ubiquitin ligand, a POI ligand, and a linker. E3 ubiquitin ligase ligands (such as VHL, MDM2, CRBN, IAPs, DCAF15, RNF4, RNF114, and DCAF16 ligands)^5–10^ are responsible for the specific recruitment of E3 ubiquitin ligases; the POI ligands are used to target and hijack the POI; and the linker molecules are used to connect the two ligands. This particular bifunctional small molecule is a powerful chemical tool that promotes POI polyubiquitination and subsequent proteasome-mediated degradation of POI by forming a stable ternary complex that drives POI in close proximity to the E3 ligase (Figure 2).^11–14^ PROTACs have many advantages over classical small molecule inhibitors (SMIs) (Figure 3).^15–19^ First, due to their unique mechanism of action (catalytic, event-driven modality), PROTACs are able to catalyse the degradation of a wide range of POI molecules. Due to this catalytic mode of action, PROTACs require much lower concentrations than SMIs to elicit the desired pharmacological effects, which may reduce the toxicity of SMIs. Second, PROTACs can target undruggable proteins. The involvement of signal transduction and transcriptional activator 3 (STAT3) in the multiple signalling pathway makes it an attractive therapeutic target; however, the lack of an obviously druggable site on the surface of STAT3 limited the development of STAT3 inhibitors. Thus, there are still no effective drugs directly targeting STAT3 approved by the Food and Drug Administration (FDA). In 2019, Shaomeng Bai et al. first developed a STAT3 PROTAC with potent biological activities *in vitro* and *in vivo.*^20^ This successful case confirms the key potential of PROTACs technology, especially in the field of undruggable targets, such as kinase p38α, and STAT3.^20^^,^^21^ Third, PROTACs can be used to overcome drug resistance caused by POI mutations. Although the mechanisms of resistance can be complex, a common mechanism is through POI mutations. In this case, the cancer cells may still depend on the target for survival and alternative strategies to drug the target may well still be efficacious. Degrading the proteins using PROTAC technology has demonstrated proof-of-principle that this strategy can overcome drug resistance. This change in the mode of action achieved by PROTACs allows resensitisation of the cancer cells. For example, PROTACs targeting mutant forms of proteins such as mutants of BCR-ABL, receptor tyrosine kinases (RTKs), and Bruton’s tyrosine kinase (BTK) have been successively reported.^22–25^ Fourth, PROTACs can overcome resistance to SMIs due to target upregulation by degrading the target. While SMIs are very effective in cancer therapy, patients often develop drug resistance and disease recurrence, consequently. PROTACs showed greater advantages in drug-resistant cancers through degrading the whole target protein. For example, asexual lymphoma kinase (ALK) PROTACs have been shown to overcome resistance to ALK inhibitors (such as alectinib, ceritinib, and brigatinib) during the treatment of non-small cell lung cancer.^26–28^ Fifth, PROTACs can improve drug selectivity and specificity. Often SMIs come with different degree of selectivity and specificity and extensive medicinal chemistry or chemical genetics efforts are needed to improve their selectivity and potency. PROTACs have been shown to be able to convert non-selective inhibitors into more selective protein degraders, which can be a potentially generalisable approach to develop selective SMIs. For example, Olson et al. developed potent and highly selective cyclin-dependent kinase 9 (CDK9) PROTACs that induce proteasome-mediated selective degradation of CDK9.^29^

As a novel approach, PROTACs have gained significant attention from academia and the pharmaceutical and biotech industries (e.g. Arvinas, Bristol Myers Squibb, C4 Therapeutics, Kymera Therapeutics, AstraZeneca, Bayer, Cullgen, Novartis, and Vertex). At present, PROTACs have been successfully employed in the degradation of different types of target proteins related to various diseases, including cancer, viral infection, immune disorders, and neurodegenerative diseases.^30–33^ Some cases reported include PROTACs targeting androgen receptor (AR) from Bristol Myers Squibb, B-cell lymphoma extra-large (BCL-X_L_) from Dialectic, BTK from Nurix, epidermal growth factor receptor (EGFR) from C4 Therapeutics, interleukin-1 receptor-associated kinase 4 (IRAK4) and STAT3 from Kymera, and tropomyosin receptor family kinases (TRK) from Cullgen. In addition, resistance caused by PROTACs was illustrated by researchers from Abbvie, and Promega reported the quantitative live-cell kinetic degradation and mechanistic profiling. Recently, ARV-110 from Arvinas, Inc., an AR-targeted PROTAC with high potency against both wild-type and mutants, exhibited satisfactory safety and tolerability in patients in a phase II clinical trial. ARV-471, an oestrogen receptor (ER) degrader from Arvinas, Inc., is also in phase II studies in women with locally advanced or metastatic ER positive/HER2 negative breast cancer.^34^^,^^35^ The alluring prospect of small molecules that remove disease protein targets from cells has spawned at least ten biotech companies. At least a half-dozen companies have brought PROTACs molecules into clinical trials (Table 1).^34^ PROTACs have opened a new chapter for the development of new drugs and novel chemical knockdown tools and brought unprecedented opportunities to the industry and academia. In this review, we will present PROTACs that target clinically validated drug targets one by one in alphabetical order of targets, according to criteria such as disease area and drug target class. We hope that this review will serve as a complementary summary to other reviews in the field of protein degradation.

**Table 1.** Selected PROTACs in and approaching the clinic.^34^^,^^35^

Agent	Company	Target	Indication	Stage
ARV-110	Arvinas	AR	Prostate cancer	Phase II
ARV-766	Arvinas	AR	Prostate cancer	Phase I
CC-94676	Bristol Myers Squibb	AR	Prostate cancer	Phase I
ARV-471	Arvinas	ER	Breast cancer	Phase II
DT2216	Dialectic	BCL-X_L_	Liquid and solid tumours	Phase I
FHD-609	Foghorn	BRD9	Synovial sarcoma	Phase I
CFT8364	C4 Therapeutics	BRD9	Synovial sarcoma, SMARCB1^-^ tumours	IND 2H2021
NX-2127	Nurix	BTK, Ikaros, Aiolos	B-cell malignancies	Phase I
NX-5948	Nurix	BTK	B-cell malignancies	IND 2H2021
KT-474	Kymera	IRAK4	Atopic dermatitis, HS	Phase I
KT-413	Kymera	IRAK4, Ikaros, Aiolos	MYD88-mutant DLBCL	IND 2H2021
CFT8919	C4 Therapeutics	EGFR^L858R^	NSCLC	IND mid-2022
KT-333	Kymera	STAT3	Liquid and solid tumours	IND 4Q2021
CG001419	Cullgen	TRK	Cancer and other diseases	IND pending

## 2. PROTACs for cancers

### 2.1. Targeting AR

Prostate cancer (PCa) is a significant cause of cancer-related death.^36^ Surgery, radiation therapy, and androgen deprivation therapies (ADTs) are first-line treatment options for patients at high risk for prostate cancer. AR signalling is critical for normal prostate development but also drives prostate cancer cell growth and survival. Previous approaches that have successfully targeted AR signalling have focussed on blocking androgen synthesis with drugs such as abiraterone and inhibiting AR function with AR antagonists such as enzalutamide and apalutamide. However, these small molecule inhibitors are ineffective against advanced prostate cancers with AR gene amplification, mutations, and alternate splicing.^37–39^

#### 2.1.1. CRBN-based PROTACs

In 2020, Scott et al. reported the CRBN-based PROTACs based on the AR antagonist enzalutamide.^40^ These PROTACs could induce the degradation of AR in a dose- and time-dependent manner. Among them, PROTAC 1 (Table 2) was a potent degradation agent, mediating 33% of AR degradation at 10 nM. Like enzalutamide, PROTAC 1 showed an inhibitory effect on the proliferation of prostate tumour cells. The discovery of enzalutamide-based PROTACs was expected to overcome the drug resistance that conventional AR antagonists bring to patients.

**Table 2.** Representative CRBN-based PROTACs targeting AR.

Compounds	Target protein	Structure	Ref.
PROTAC 1	AR	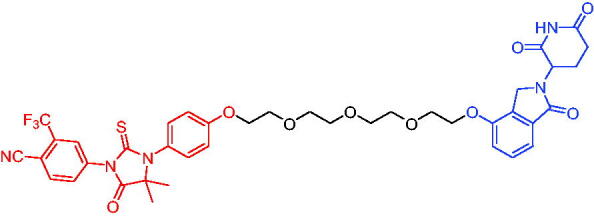	^40^
PROTAC 2	AR	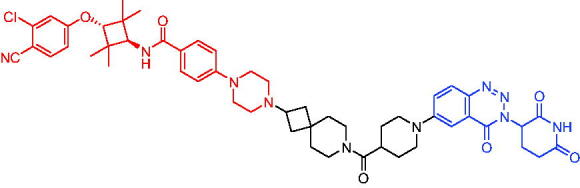	^41^
PROTAC 3	AR	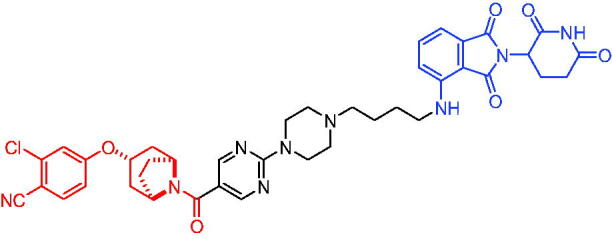	^42^
PROTAC 4	AR	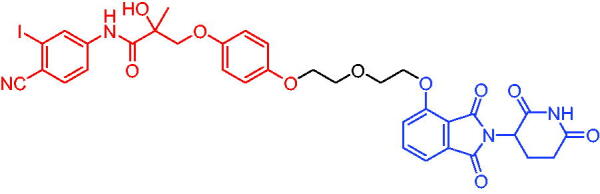	^43^
PROTAC 5	AR	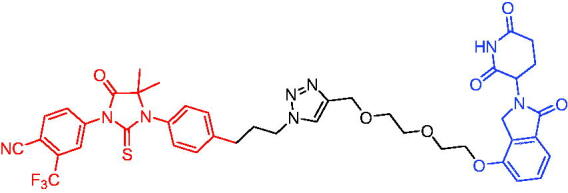	^44^
PROTAC 6	AR	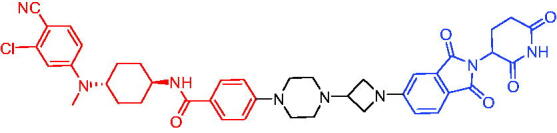	^45^
PROTAC 7	AR	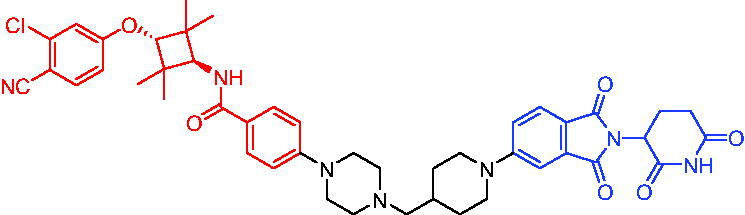	^46^

In the same year, Takwale et al. disclosed some new AR PROTACs for the treatment of metastatic castration-resistant prostate cancer (CRPC).^41^ Primarily, they utilised TD-106 (a novel CRBN ligand) as an E3 ligase ligand. Among the new CRBN-based PROTACs, PROTAC 2 (Table 2) effectively degraded AR protein with a degradation concentration 50% of 12.5 nM and maximum degradation of 93% in LNCaP prostate cancer cells. Moreover, PROTAC 2 showed good liver microsomal stability and *in vivo* pharmacokinetic properties.

In 2021, Chen et al. designed and synthesised a new series of CRBN-based PROTACs using newly discovered AR antagonists.^42^ The cell inhibitions for all of these synthetic compounds in AR + VCaP cell lines at different concentrations were tested. The representative compound, PROTAC 3 (Table 2), effectively inhibited 50.44% of cell liability at 1.0 μM. The authors believed that the discovery of the above AR PROTACs provided further ideas for the development of novel drugs for the treatment of prostate cancer.

In order to find PROTACs with lower toxicity and better binding affinity than before, another set of CRBN-based PROTACs consisting of bicalutamide and thalidomide were designed, synthesised, and biologically evaluated.^43^ The novel AR PROTACs had their abilities to induce AR degradation. In particular, PROTAC 4 (Table 2) was shown to significantly induce AR degradation in a dose- and time-dependent manner.

The novel heterobifunctional AR PROTACs based on the high-affinity AR agonist RU59063 connected through a 1,2,3-triazole linker to a CRBN ligand were reported by Liang et al. in 2021.^44^ The novel synthesised AR PROTACs displayed moderate to satisfactory AR binding affinity and might lead to antagonist activity against AR. As a representative compound, PROTAC 5 (Table 2) could potently degrade AR. Moreover, due to the strong fluorescence properties of pomalidomide derivatives, AR PROTACs were found to be effectively internalised and visualised in LNCaP (AR+) cells. In addition, the molecular docking of PROTAC 5 with AR and the active site of DDB1-CRBN E3 ubiquitin ligase complex provided guidance to design new PROTAC degrons targeting AR for prostate cancer therapy.

Xiang et al. described some AR PROTACs using the CRBN ligand, thalidomide, and different classes of AR antagonists.^45^ PROTAC 6 (Table 2) achieved picomolar DC_50_ values and >98% of D_max_ in the VCaP cell line with a wild-type AR and in the LNCaP cell line carrying a T878A-mutated AR mutant. Moreover, PROTAC 6 reduced AR protein by >80% at 0.1 nM in the 22Rv1 cell line carrying an AR-V7 variant and at 1 nM in the MDA-PCa-2b cell line carrying a double AR mutation. PROTAC 6 potently inhibited cell growth with IC_50_ values of 1.5 and 16.2 nM in the VCaP and LNCaP AR + prostate cancer cell lines, respectively. It displayed excellent PK parameters with both intravenous and oral routes of administration in mice and achieves extensive tissue distribution. Oral administration of PROTAC 6 effectively reduced AR protein in the VCaP xenograft tumour tissue in mice and inhibits VCaP tumour growth. Their data demonstrated that PROTAC 6 was a promising AR degrader in further extensive evaluations for the treatment of AR + prostate cancer and other human diseases in which AR plays a key role.

Subsequently, Han et al. also reported the design, synthesis, and evaluation of new AR PROTACs using a potent AR antagonist and thalidomide with the objective of discovering potent and orally bioavailable AR PROTACs.^46^ Employing thalidomide to recruit cereblon/cullin 4 A E3 ligase and through the rigidification of the linker, they discovered highly potent AR PROTACs with good oral pharmacokinetic properties in mice with PROTAC 7 (Table 2) being the best compound (DC_90_ = 3.5 nM). PROTAC 7 achieved 67% oral bioavailability in mice, effectively reduced AR protein and suppresses AR-regulated genes in tumour tissues with oral administration, leading to the effective inhibition of tumour growth in mice without signs of toxicity. Their research supported the development of an orally active AR PROTAC for the treatment of prostate cancer and provided insights and guidance into the design of orally active PROTACs.

#### 2.1.2. VHL-based PROTACs

In 2018, Salami et al. reported the first series of VHL-based AR PROTACs through connecting enzalutamide and a VHL ligand with distinct linkers.^47^ The potent PROTAC, PROTAC 8 (Table 3), was a low-nanomolar AR degrader able to degrade about 95% of cellular AR proteins. PROTAC 8 had an inhibitory proliferative effect on prostate tumour cells and degraded clinically relevant AR mutants. Furthermore, PROTAC 8 reduced AR levels in prostate cancer-resistant cells LNCaP (approximately 3.5-fold at 10 μM), while AR was substantially increased in cells treated with enzalutamide (approximately 17.5-fold at 10 μM). PROTAC 8 demonstrated that protein degradation could address the drug resistance barrier of enzalutamide.

**Table 3.** Representative VHL-based PROTACs targeting AR.

Compounds	Target protein	Structure	Ref.
PROTAC 8	AR	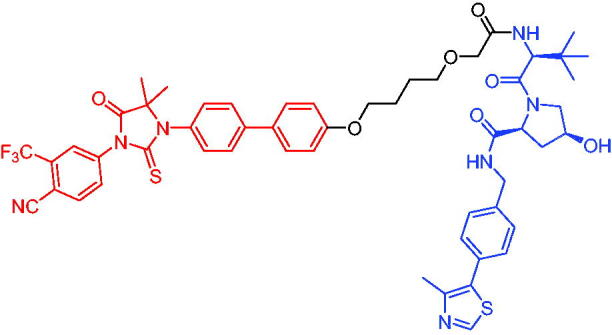	^47^
PROTAC 9	AR	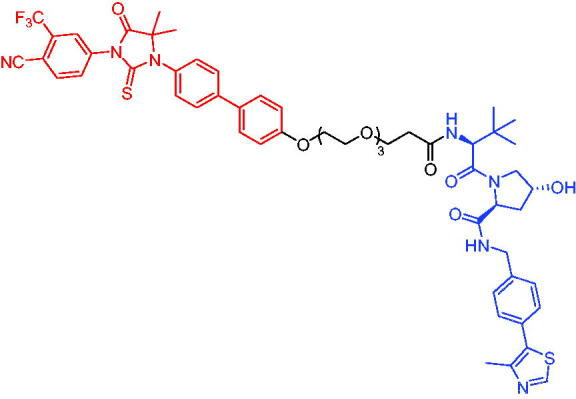	^38^
PROTAC 10	AR	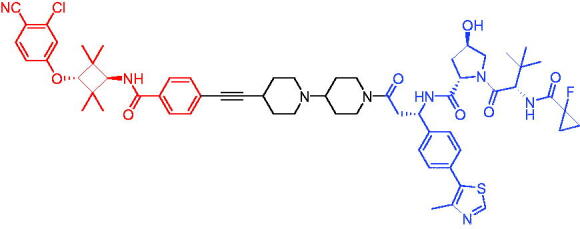	^48^
PROTAC 11	AR	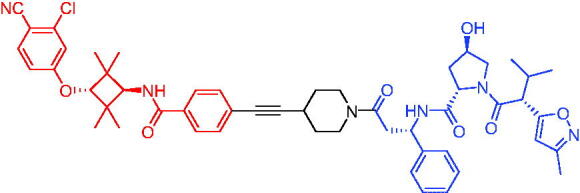	^49^
PROTAC 12	AR	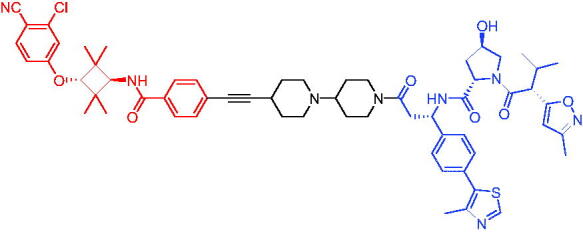	^50^
PROTAC 13	AR	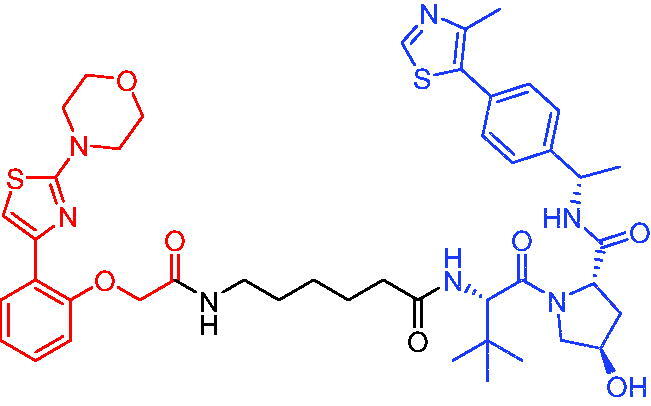	^51^
PROTAC 14	AR	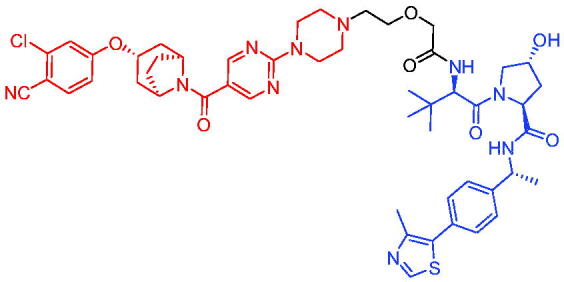	^42^
PROTAC 15	AR	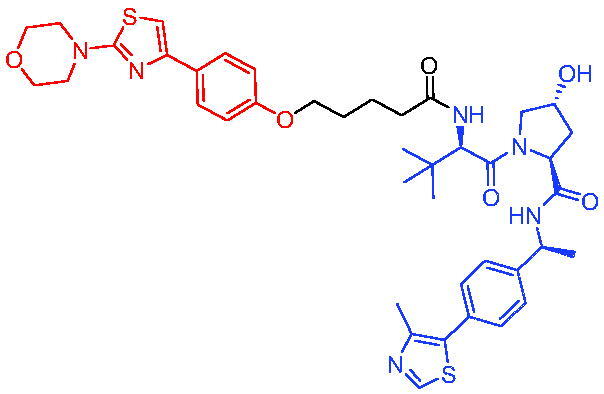	^52^

Subsequently, Kahn et al. developed some AR targeting PROTACs.^38^ Like CRBN ligands, VHL ligands have been successfully used for the design of AR targeting PROTACs. As with PROTAC 8, PROTAC 9 (Table 3) was also effective in inducing the degradation of AR protein. The authors believed that by better understanding the link between PROTACs structure and cellular efficacy, they would be able to rationalise the design of better molecules and more effectively translate PROTACs molecules into the clinic.

In 2019, Han et al. reported their discovery of potent small-molecule AR PROTACs.^48^ PROTAC 10 (Table 3) was effective in inducing AR degradation at concentrations lower than 1 nM in LNCaP and VCaP prostate cancer cell lines with a 24 h treatment time and was capable of achieving complete AR degradation in these cell lines. PROTAC 10 effectively suppressed AR-regulated gene expression in a dose-dependent manner and was effective at concentrations as low as 10 nM in the LNCaP and VCaP cell lines with 24 h treatment time. It potently inhibited cell growth in the LNCaP, VCaP, and 22Rv1 prostate cancer cell lines and was >100 times more potent than the two AR antagonists that were tested. A single dose of PROTAC 10 also effectively reduced AR and PSA proteins in VCaP xenograft tumour tissues in mice for more than 48 h. Taken together, their data demonstrated that PROTAC 10 was an extremely potent AR degrader.

In the same year, Han et al. reported their design, synthesis, and biological characterisation of new highly potent small-molecule AR PROTACs using a potent AR antagonist and E3 ligase ligands with weak binding affinities to VHL protein.^49^ Their study resulted in the discovery of PROTAC 11 (Table 3), which effectively induced degradation of AR protein in AR + LNCaP, VCaP, and 22Rv1 prostate cancer cell lines with DC_50_ values of 0.2–1 nM. PROTAC 11 was capable of reducing the AR protein level by >95% in these AR + prostate cancer cell lines and effectively reduced AR-regulated gene expression suppression. For the first time, they demonstrated that an E3 ligand with micromolar binding affinity to its E3 ligase complex could be successfully employed for the design of highly potent and efficient PROTACs and their finding might have a significant implication for the field of PROTACs research.

By further optimisation of PROTAC 10 and PROTAC 11, Shaomeng Zhao et al. designed and synthesised another series of AR PROTACs in 2020.^50^ The representative compound, PROTAC 12 (Table 3), potently degraded AR in AR + breast cancer cell lines and was much more potent than enzalutamide in inhibition of cell growth and induction of cell cycle arrest and/or apoptosis. Moreover, PROTAC 12 effectively and completely degraded AR protein in xenograft tumour tissue and was more effective than enzalutamide in achieving tumour growth inhibition in the MDA-MB-453 xenograft model in mice. The authors concluded that this study provided a strong preclinical rationale for the development of AR PROTACs to treat AR + human breast cancer.

In 2021, Lee et al. developed a novel AR degrader for overcoming resistance to second-line antiandrogen therapy (SAT) in patients with CRPC by conjugating ligands of VHL and AR.^51^ PROTAC 13 (Table 3) could induce AR-V7 and AR-FL degradation with DC_50_ values of 0.37 and 2 µM respectively. PROTAC 13 inhibited CaP cellular proliferation and increased apoptosis only in androgen-responsive CaP cells. When resistant cells were treated with PROTAC 13, decreased cellular proliferation and reduced tumour growth were observed both *in vitro* and *in vivo*. Together, these results suggested that PROTAC 13 was a novel small-molecule degrader that might be effective against SAT-resistant CRPC by degrading AR-V7 and AR-FL.

In 2021, Chen et al. reported success in the development of VHL-based AR PROTACs by optimising AR antagonists and E3 ligase ligands that potently induced the degradation of AR.^42^ As a potent AR degrader, PROTAC 14 (Table 3) could induce the degradation of AR protein in VCaP cell lines in a time-dependent manner, achieving the IC_50_ value of less than 0.25 μM. PROTAC 14 was five times less toxic than EZLA and worked with an appropriate half-life (t_1/2_) or clearance rate. Also, it had a significant inhibitory effect on tumour growth in zebrafish transplanted with VCaP. Therefore, PROTAC 14 provided a further idea of developing novel drugs for prostate cancer.

The AR-V7 splice variant has been characterised extensively and current clinical trials in CRPC are exploring the use of AR-V7 as a biomarker. New therapeutic molecules that selectively target AR-V7 are also being explored. However, there is a dearth of information available on the selectivity, phenotypic responses in AR-V7 dependent cell lines, and pharmacokinetic properties of such molecules. Using proprietary computational algorithms and rational SAR optimisation, Bhumireddy et al. developed a selective AR-V7 degrader, PROTAC 15 (Table 3) with DC_50_ of 0.32 µM by recruiting VHL E3 ligase to AR DBD binder.^52^ This molecule effectively degraded AR-V7 in a CRPC cell line and demonstrated good oral bioavailability in mouse PK studies. This tool compound can be used to evaluate the pharmacological effects of AR-V7 degraders. Further exploration of SAR could be pursued to develop more optimised lead compounds.

#### 2.1.3. IAP-based PROTACs

Derived from IAP ligands, a series of novel IAP-based PROTACs targeting AR were developed by Shibata et al. in 2018.^53^ Among them, PROTAC 16 (Table 4) showed effective protein knockdown activity against AR. Consistent with the degradation of the AR protein, PROTAC 16 inhibited AR-mediated gene expression and proliferation of androgen-dependent prostate cancer cells. In addition, PROTAC 16 efficiently induced caspase activation and apoptosis in prostate cancer cells, which was not observed in the cells treated with AR antagonists. These results suggested that PROTAC 16 could be lead for an anticancer drug against prostate cancers that exhibited AR-dependent proliferation.

**Table 4.** Representative IAP-based PROTAC targeting AR.

Compound	Target protein	Structure	Ref.
PROTAC 16	AR	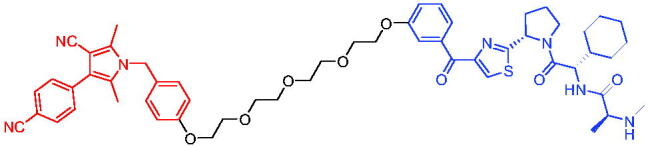	^53^

### 2.2. Targeting BCL-X_L_

BCL-X_L_ is one of the important proteins in the B-cell lymphoma 2 family, which plays a pivotal role in controlling the life-cycle of cell *via* regulating the intrinsic apoptotic pathway.^54^ BCL-X_L_ is a very important cancer target. Inhibition of these BCL-2 family proteins with inhibitors has been extensively studied as a strategy for cancer treatment, resulting in the identification of ABT263 (navitoclax, a dual BCL-2 and BCL-X_L_ inhibitor), ABT199 (venetoclax, a BCL-2 selective inhibitor), and several BCL-X_L_ and MCL-1 monoselective inhibitors are promising anticancer drug candidates.^55^ To date, ABT199 is the only antitumour agent approved by the Food and Drug Administration (FDA) that targets BCL-2 family proteins. ABT263 is not approved because inhibition of BCL-X_L_ induces target and dose-limiting thrombocytopenia.

#### 2.2.1. CRBN-based PROTACs

In 2020, He et al. disclosed the first ABT263-based PROTAC for BCL-X_L_.^56^ The most promising degradation agent, PROTAC 17 (Table 5), mediated a significant decrease in BCL-X_L_ (DC_50_ = 46 nM, D_max_ = 96.2%). Unlike ABT263, PROTAC 17 was less toxic to platelets. With further improvements, PROTACs targeting BCL-X_L_ had the potential to become safer and more effective haemolysis agents than BCL-X_L_ inhibitors.

**Table 5.** Representative CRBN-based PROTACs targeting BCL-X_L_.

Compounds	Target protein	Structure	Ref.
PROTAC 17	BCL-X_L_	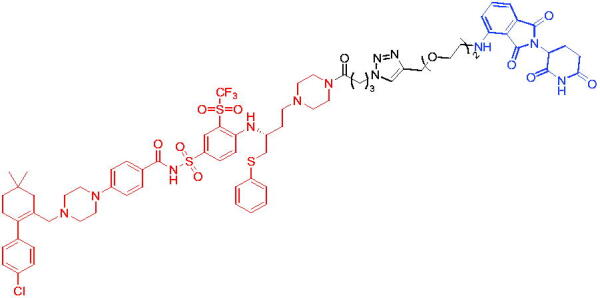	^56^
PROTAC 18	BCL-X_L_	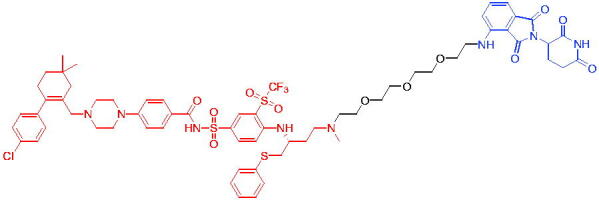	^57^

In the same year, Zhang et al. described another series of BCL-X_L_ based PROTACs by conjugating ABT-263 and a CRBN ligand.^57^ Most of BCL-X_L_ based PROTACs were more potent in killing cancer cells than their parent compound ABT-263. The most active BCL-X_L_ degrader, PROTAC 18 (Table 5), was 20 times more potent than ABT-263 against MOLT-4 T-ALL cells and 100 times more selective than human platelets against MOLT-4 cells.

#### 2.2.2. VHL-based PROTACs

In 2020, Khan et al. developed some potent and specific BCL-X_L_ degraders that showed great *in vivo* therapeutic potential for cancer.^58^ All BCL-X_L_ degraders were developed on the basis of ABT263. Representative PROTAC 19 (Table 6) could degrade effectively BCL-X_L_. PROTAC 19 was effective in inhibiting the growth of several xenogeneic tumours *in vivo* when used as a single agent or in combination with other chemotherapeutic agents without causing significant thrombocytopenia. These findings suggested the potential to use PROTACs strategy to reduce the toxicity of target drugs and rescue the therapeutic potential of previously untreatable targets. In addition, PROTAC 19 could be developed as a safe first-in-class anticancer agent against BCL-X_L_.

**Table 6.** Representative VHL-based PROTACs targeting BCL-X_L_.

Compounds	Target protein	Structure	Ref.
PROTAC 19	BCL-X_L_	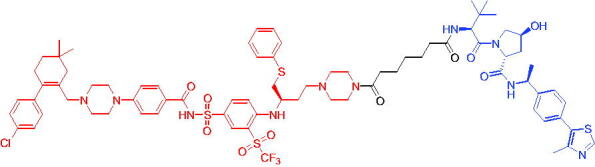	^58^
PROTAC 20	BCL-X_L_	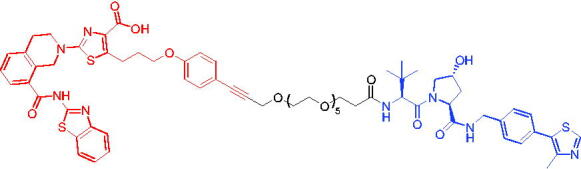	^59^
PROTAC 21	BCL-X_L_	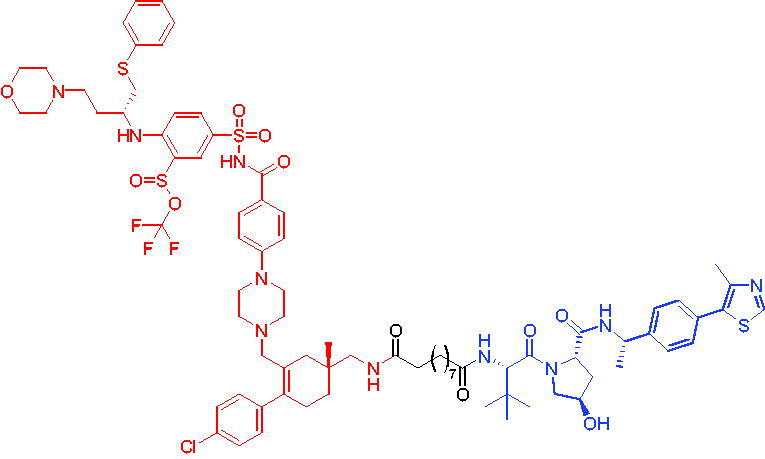	^60^

To date, no ternary complex structures of BCL-X_L_ with a PROTAC and an E3 ligase have been successfully determined. In 2020, Chung et al. reported the design, characterisation, and X-ray structure of a VHL E3 ligase-recruiting BCL-X_L_ PROTAC degrader.^59^ The representative degrader PROTAC 20 (Table 6) consisted of BCL-X_L_ antagonist A-1155463 and VHL E3 ligase binder, which could selectively degrade BCL-X_L_ with the DC_50_ value of 4.8 nM in THP-1 cells line. This work illustrated the challenges associated with the rational design of bifunctional molecules where interactions involved composite interfaces.

In 2021, Pal et al. designed and synthesised a novel BCL-X_L_ targeting degrader (PROTAC 21, Table 6) based on BCL-X_L_/BCL-2 dual inhibitor ABT-263 by tethering the pro-*R* methyl group on the cyclohexene ring of ABT-263.^60^ PROTAC 21 could induce effective degradation of BCL-X_L_. PROTAC 21 also appeared to potently inhibit BCL-2 through the formation of stable {BCL-2: PROTAC 21: VCB} ternary complexes in live cells. PROTAC 21 possessed a unique mechanism of action (MOA) in inhibiting antiapoptotic BCL-2 proteins, i.e. potent degradation of BCL-X_L_ and simultaneously enhanced inhibition of BCL-2, that enabled its high potency against BCL-X_L_ dependent, BCL-2 dependent, and BCL-X_L_/BCL-2 dual-dependent cancer cells. This was the first time that such a hybrid mechanism had been observed in PROTACs.

#### 2.2.3. IAP-based PROTACs

To overcome mechanism of resistance, PROTACs based on recruiting alternative E3 ligases could be generated. In 2020, Zhang et al. described a series of PROTACs that recruit IAP E3 ligases for BCL-X_L_ degradation.^61^ PROTAC 22 (Table 7) efficiently induced BCL-X_L_ degradation in malignant T-cell lymphoma cell line MyLa 1929. Furthermore, compared with ABT-263, PROTAC 22 showed comparable cell killing effects in MyLa 1929 cells whereas the on-target platelet toxicity was significantly reduced. In addition, PROTAC 22 powerfully degraded BCL-X_L_ in multiple cancer cell lines, suggesting that BCL-X_L_ PROTACs had considerable potential for application in cancer therapy.

**Table 7.** Representative IAP-based PROTAC targeting BCL-X_L_.

Compound	Target protein	Structure	Ref.
PROTAC 22	BCL-X_L_	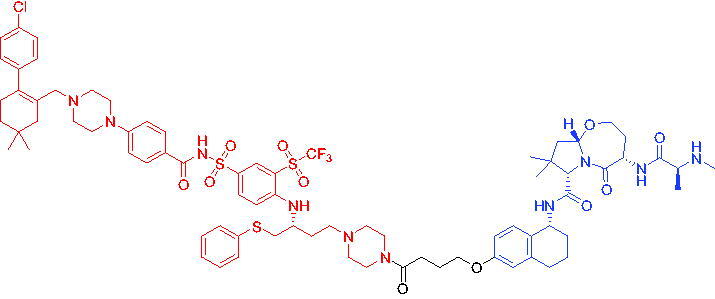	^61^

### 2.3. Targeting BRD9

BRD9 is the bromodomain-containing subunit of the BAF (BRG-/BRM-associated factor) and its close homolog BRD7 is the subunit of PBAF (polybromo-associated BAF). BAF and PBAF are two variants of the SWI/SNF complex, which regulate gene expression, DNA replication, and DNA repair.^62^ Overexpression of BRD9 has been found in some cancers such as cervical cancer. BRD9 is an important target in cancer therapy.

#### 2.3.1. CRBN-based PROTACs

In 2017, the first PROTAC targeting BRD9 was developed by Remillard et al. The PROTAC was conjugated with BRD9 inhibitor and pomalidomide.^63^ PROTAC 23 (Table 8) showed a dose-dependent degradation of BRD9. It had a significant selectivity for BRD9 over BRD4 and BRD7. Compared to small-molecule inhibitors, PROTAC 23 exhibited 10 to 100-fold potency in degrading BRD9 with DC_50_ and IC_50_ values of 50 nM and 104 nM, respectively. BRD9-based PROTACs could be a potential strategy for the treatment of human acute leukaemia.

**Table 8.** Representative CRBN-based PROTACs targeting BRD9.

Compounds	Target protein	Structure	Ref.
PROTAC 23	BRD9	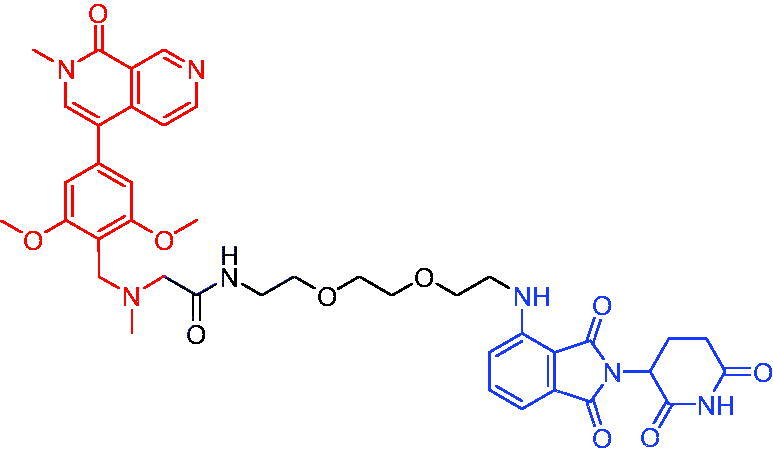	^63^
PROTAC 24	BRD9	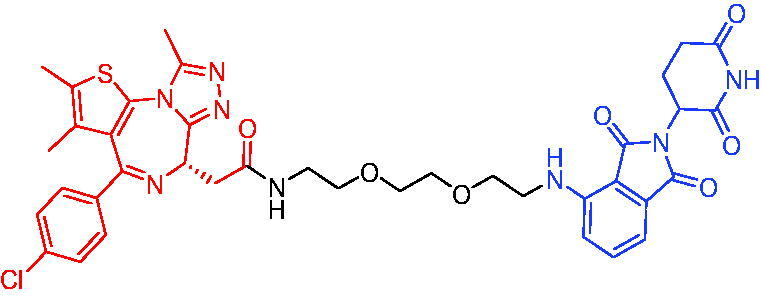	^64^

To investigate the pharmacokinetic properties of BRD9 PROTACs molecules, Goracci et al. described a study on the metabolism of a series of BET PROTACs in cryopreserved human hepatocytes at multiple time points.^64^ The results indicated that linkers’ chemical nature and length of PROTAC 24 (Table 8) played a major role in pharmacokinetic properties. To further interpret the data, a number of BRD9 PROTACs were also tested for metabolism by human cytochrome P450 3A4 (CYP3A4) and human aldehyde oxidase (hAOX).

#### 2.3.2. VHL-based PROTACs

The first VHL-based PROTAC of BRD7/9 was developed by Zoppi et al. in 2019. Based on the BRD7/9 ligand, BRD7/9 PROTAC was constructed that induced the degradation of BRD7/9 in the presence of VHL E3 ubiquitin ligase.^65^ PROTAC 25 (Table 9) caused both degradation of BRD7 (DC_50_ = 4.5 nM) and BRD9 (DC_50_ = 1.8 nM). In addition, PROTAC 25 showed cytotoxic effects in EOL-1 (acute myeloid eosinophilic leukaemia) and A-204 (malignant rhabdoid tumour) cell lines, with EC_50_ values of 3 nM (EOL-1) and 40 nM (A-402), respectively. These findings qualified a new chemical tool for BRD7/9 knockdown and provided a roadmap for PROTAC development against seemingly incompatible combinations of target ligases.

**Table 9.** Representative CRBN-based PROTAC targeting BRD7/9.

Compound	Target protein	Structure	Ref.
PROTAC 25	BRD7/9	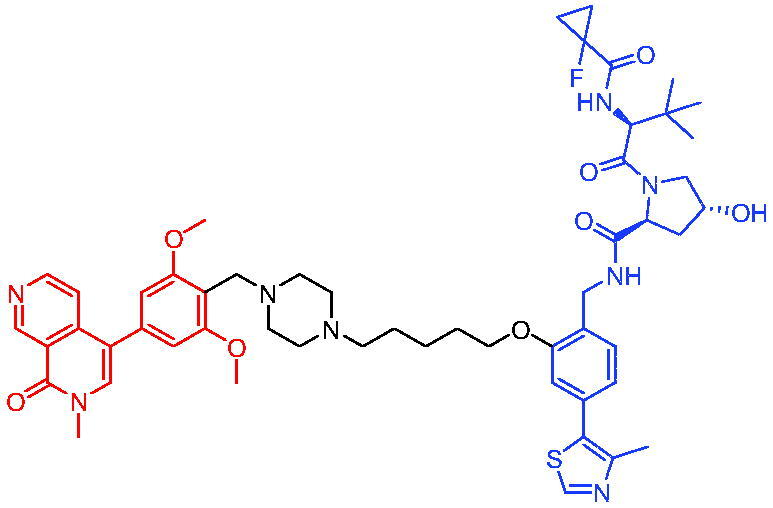	^65^

### 2.4. Targeting BTK

B-cell receptor (BCR) is an important regulator in B-cell signalling in adhesion, survival, and growth. For BCR pathway, BTK is indispensable since it worked as a membrane proximal signal molecule for the activation and proliferation of B cell.^66–69^ Inhibition of BTK kinase activity has been shown to be an important and practical approach for the treatment of non-Hodgkin’s lymphoma (NHL). Ibrutinib is a class of covalent BTK inhibitors approved by the FDA for the treatment of several types of NHL. However, due to a missense mutation in BTK C481S, NHL patients have developed drug resistance after treatment with ibrutinib. Ibrutinib also lost the inhibitory effect on NHL tumour cell growth caused by the BTK C481S mutation.^70^

#### 2.4.1. CRBN-based PROTACs

In 2018, degradation of BTK mutants by PROTACs for potential treatment of ibrutinib-resistant non-Hodgkin lymphomas, Sun et al. first reported two novel sets of BTK PROTACs for degrading drug-resistant BTK.^25^^,^^71^ Among them, PROTAC 26 (Table 10) had the ability to degrade different C481 BTK mutants with DC_50_ values below 50 nM. PROTAC 26 showed better growth inhibition of wild-type BTK cells than ibrutinib. In a mouse xenograft model inoculated with C481S BTK HBL-1 cells, PROTAC 26 promoted rapid tumour regression, with 36% and 63% tumour reduction at 30 or 100 mg/kg, respectively. The above results suggested that the BTK PROTAs provided the great potential of inhibiting the BTK functions, especially for ibrutinib-resistant lymphomas.

**Table 10.** Representative CRBN-based PROTACs targeting BTK.

Compounds	Target protein	Structure	Ref.
PROTAC 26	BTK	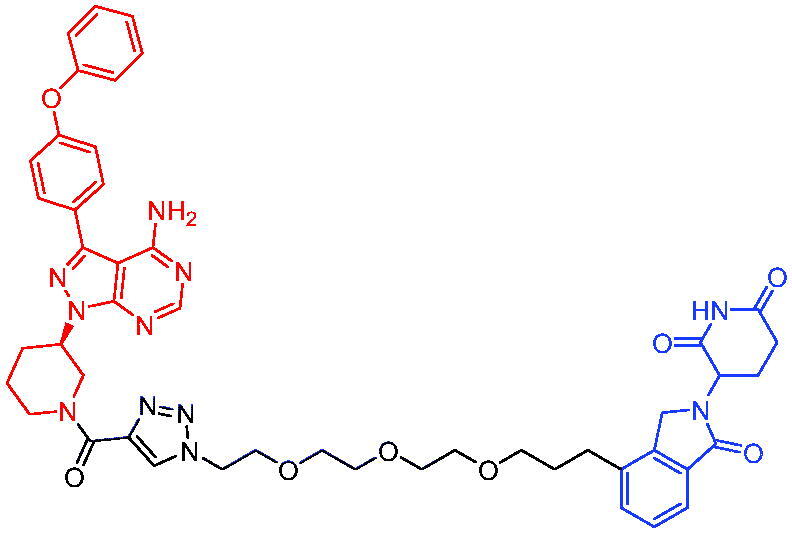	^25^ ^,^ ^71^
PROTAC 27	BTK	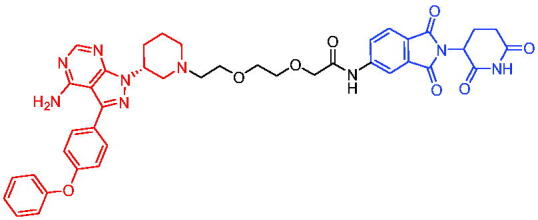	^72^
PROTAC 28	BTK	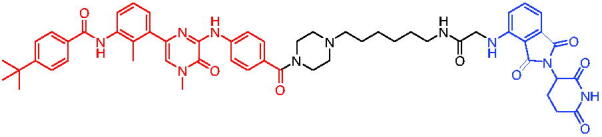	^73^
PROTAC 29	BTK	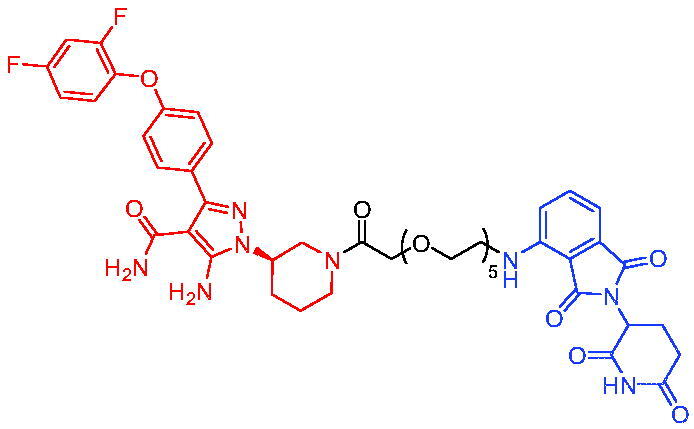	^74^
PROTAC 30	BTK	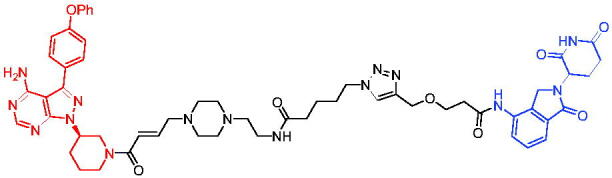	^75^
PROTAC 31	BTK	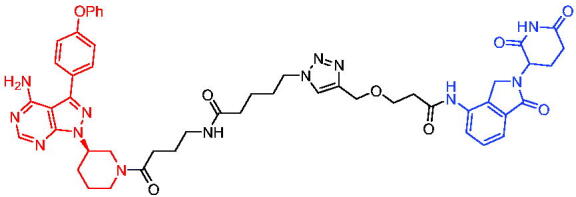	^75^
PROTAC 32	BTK	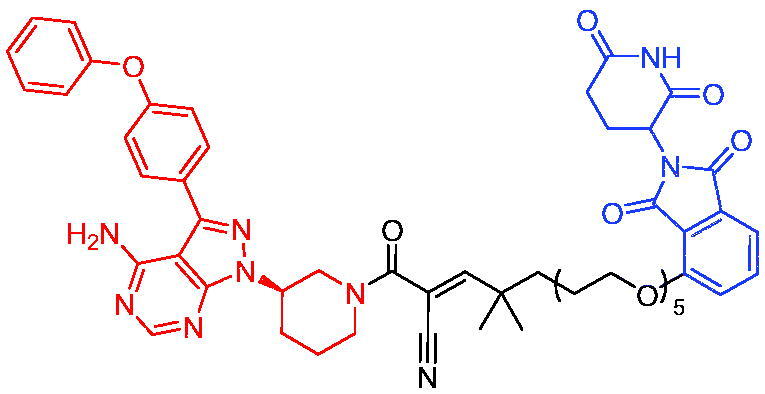	^76^
PROTAC 33	BTK	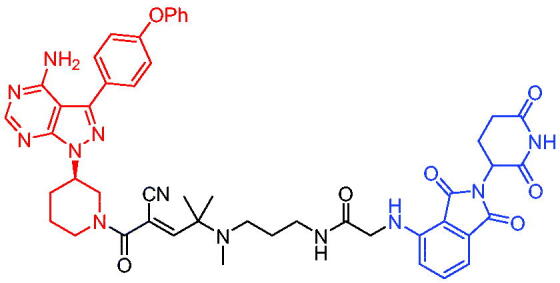	^77^
PROTAC 34	BTK	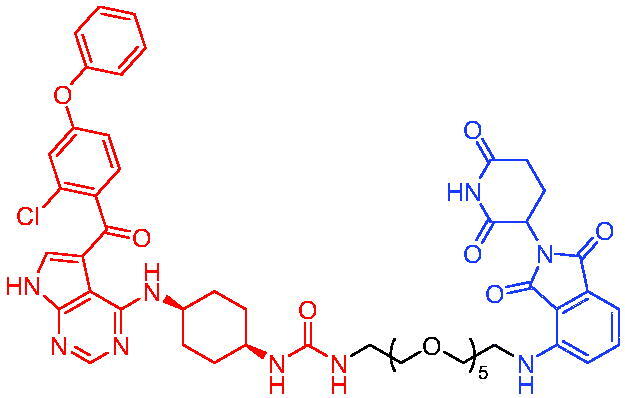	^78^

In the same year, Buhimschi et al. developed another novel ibrutinib-based BTK PROTAC.^72^ For wild-type and C481S BTK, PROTAC 27 (Table 10) effectively induced BTK degradation, with DC_50_ of 14.6 nM and 14.9 nM, respectively.

At almost the same time, a more specific BTK PROTAC named DD-04–015 was disclosed, which showed BTK degradation in a dose- and time-dependent way.^73^ After further optimisation, a new degrader PROTAC 28 (Table 10) with stronger ability to degrade C481S-BTK was developed. Compared with DD-04–015, PROTAC 28 showed a strong antiproliferation inhibition with an IC_50_ of 5.1 nM against nested cell lymphoma (MCL) cells *in vitro* and an efficient anticancer effect *in vivo*.

Zorba et al. also produced the PROTACs targeting BTK by conjugation of phenyl-pyrazole to pomalidomide.^74^ Among the reported degraders, PROTAC 29 (Table 10) induced the rapid degradation of BTK with a DC_50_ of 5.9 ± 0.5 nM after 24 h of treatment in Ramos cells. When evaluated *in vivo*, efficient BTK degradation was also observed in the lung and spleen in the BTK degrader-treated rats.

In 2019, Tinworth et al. researched the effect of covalent binding on PROTAC-mediated BTK degradation by preparing covalently bound and reversibly bound PROTACs from the covalent BTK inhibitor ibrutinib.^75^ They found that covalently bound PROTAC (PROTAC 30, Table 10) inhibited BTK degradation, while reversibly bound PROTAC (PROTAC 31, Table 10) promoted BTK degradation. They concluded that catalysis was essential for successful PROTAC-mediated degradation.

In 2020, Gabizon et al. developed a reversible covalent BTK degrader, PROTAC 32 (Table 10), which consisted of the BTK inhibitor ibrutinib and a thalidomide derivative.^76^ PROTAC 32 showed specific and remarkable potency on BTK degradation with DC_50_ value of less than 10 nM and D_max_ near 90% in Mino cells. Compared to the irreversible PROTACs, PROTAC 32 presented a better potency and selectivity in BTK application.

In 2020, Guo et al. reported a unique bifunctional BTK degrader.^77^ The promising compound PROTAC 33 (Table 10) could reduce approximately 81% of endogenous BTK protein at 0.2 μM. Unlike other PROTACs that had low target occupancy due to poor permeability, PROTAC 33 had high target occupancy and acted as both an inhibitor and a degrader. Compared to other reported BTK degraders, PROTAC 33 outperformed in cell survival and target exposure assays and has a reasonable plasma half-life for *in vivo* application. The authors believed that this work would not only help to develop optimal BTK degraders for clinical applications, but also provided a strategy for treating tumours.

In 2021, Zhao et al. discovered a series of novel BTK PROTACs based on the reversible non-covalent BTK inhibitor ARQ531.^78^ Both the weak and strong binding warhead based PROTACs could degrade BTK^WT^ and BTK^C481S^, but strong binding warhead based PROTACs are more potent on BTK^C481S^ TMD8 cell proliferation inhibition. PROTAC 34 (Table 10) was the most potent PROTAC with strong BTK^WT^ and BTK^C481S^ degradation (DC_50_ = 41.9 Nm, D_max_ = 93.0%), effectively BTK^WT^ and BTK^C481S^ TMD8 cell proliferation inhibition (IC_50_ = 253.5 nM), moderate membrane permeability and good plasma stability. These data provided a basis for developing new and potent reversible non-covalent PROTAC-based therapeutic molecules.

#### 2.4.2. IAP-based PROTACs

In 2018, some BTK PROTACs were designed and synthesised by Zorba et al.^74^ The PROTACs were developed through the conjugation of a BTK inhibitor and IAP ligand. The authors found that BTK degradation was inefficient when either IAP or VHL are recruited instead of CRBN. The representative compound, PROTAC 35 (Table 11), was shown in Table 1.

**Table 11.** Representative CRBN-based PROTACs targeting BTK.

Compounds	Target protein	Structure	Ref.
PROTAC 35	BTK	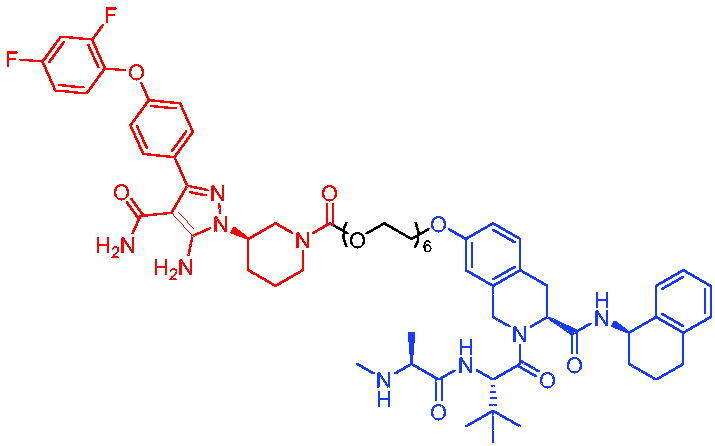	^74^
PROTAC 36	BTK	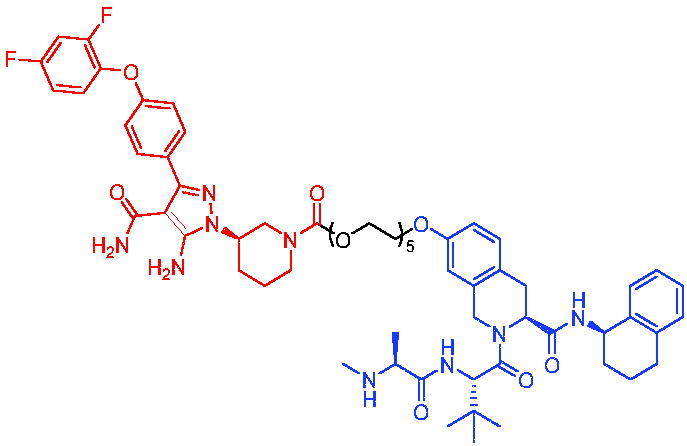	^79^
PROTAC 37	BTK	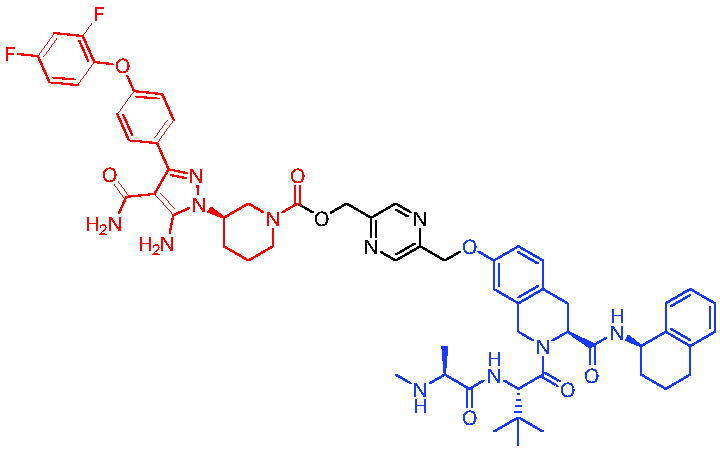	^79^

Using HSQC NMR and computational models, Schiemer et al. designed and synthesised two new BTK PROTACs (PROTAC 36 and PROTAC 37, Table 11) linking aminopyrazole derivatives to IAP ligands in 2020.^79^ PROTAC 36 showed BTK degradation in a dose- and time-dependent way with a DC_50_ of 182 ± 57 nM. This degradation was rescued after shortening the pentameric glycol linker to a non-permissive dimeric glycol linker (PROTAC 37), consistent with the mechanism of action of protein degrader.

### 2.5. Targeting EGFR

EGFR is a glycoprotein with tyrosine kinase activity that is involved in tumour cell proliferation, angiogenesis, tumour invasion, metastasis, and apoptosis inhibition. EGFR overexpression plays an important role in the development of malignant tumours, such as glioblastoma, NSCLC, breast cancer, head and neck cancer, pancreatic cancer.^80–85^ After decades of development, many EGFR inhibitors have emerged. Despite great therapeutic successes, the clinical use of these EGFR inhibitors inevitably leads to acquired resistance, which presents new challenges for cancer treatment.^86^

#### 2.5.1. CRBN-based PROTACs

In 2020, Zhang et al. reported some EGFR degraders based on the fourth-generation EGFR inhibitor pyrido[*3,4-d*] pyrimidine and a CRBN ligand.^87^ They found that all the degraders were capable of inducing EGFR degradation. For example, PROTAC 38 (Table 12) induced EGFR degradation with a DC_50_ = 45.2 nM in HCC827 cells. PROTAC 38 could significantly induce the apoptosis of HCC827 cells and arrest the cells in G1 phase. Further evaluation of PROTAC 38’s activity in degrading EGFR was ongoing, and data would be disclosed in due course.

**Table 12.** Representative CRBN-based PROTACs targeting EGFR.

Compounds	Target protein	Structure	Ref.
PROTAC 38	EGFR	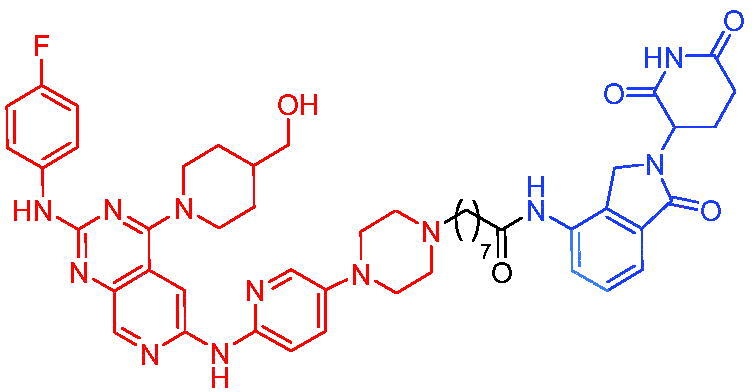	^87^
PROTAC 39	EGFR	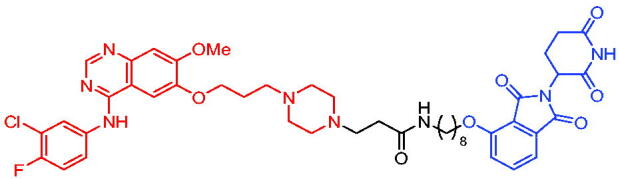	^88^
PROTAC 40	EGFR	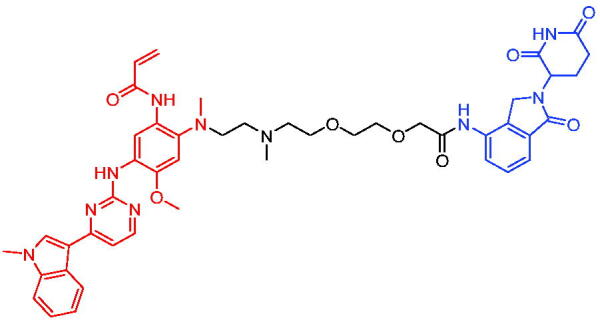	^89^
PROTAC 41	EGFR	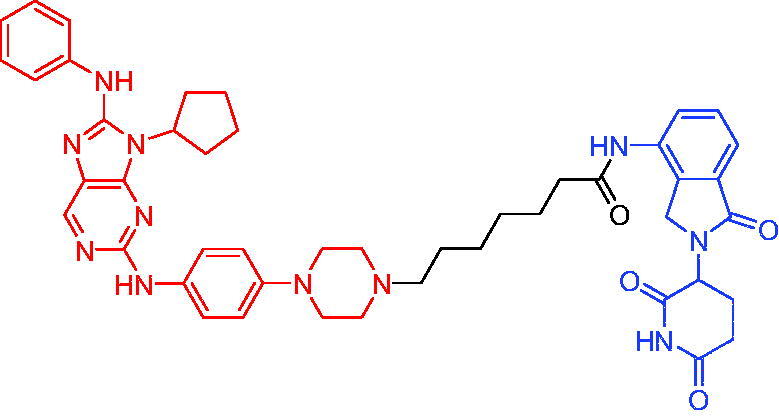	^90^
PROTAC 42	EGFR	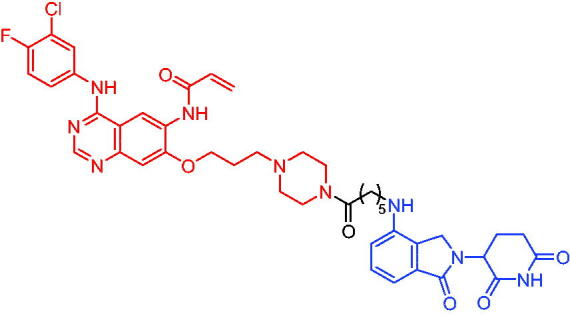	^91^
PROTAC 43	EGFR	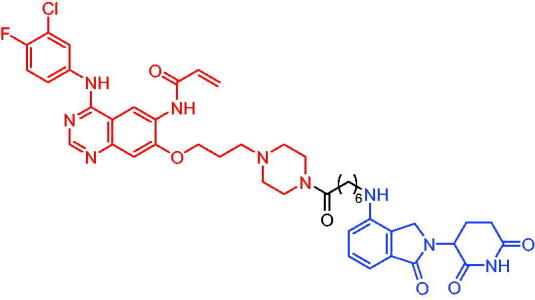	^91^
PROTAC 44	EGFR	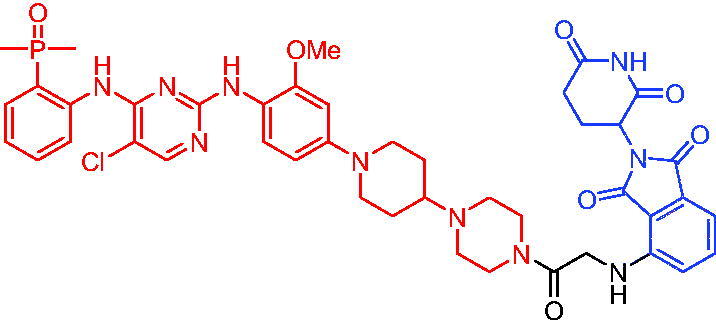	^92^
PROTAC 45	EGFR	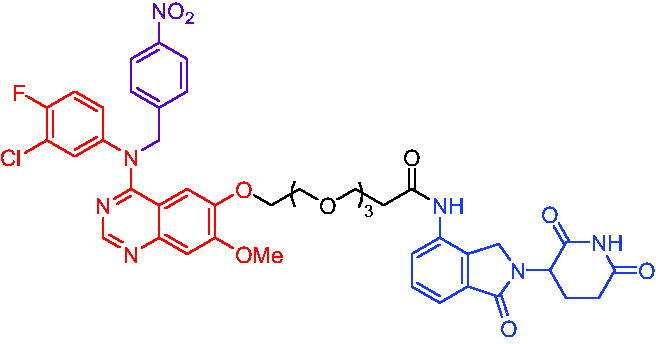	^93^

In 2020, PROTAC 39 (Table 12), consisting of gefitinib and thalidomide, was documented by Cheng et al. as an EGFR degrader.^88^ PROTAC 39 induced obvious degradation of mutant EGFR in lung cancer cells. PROTAC 39 was more potent than the previously reported EGFR degraders. Moreover, PROTAC 39 inhibited cell proliferation more effectively compared to the parent drug gefitinib.

Immediately after, He et al. disclosed a highly potent EGFR degrader called PROTAC 40 (Table 12).^89^ PROTAC 40 induced efficient EGFR degradation in PC9 cells. In addition, PROTAC 40 showed good inhibitory effects on PC9 cells and H1975 cells with corresponding IC_50_ values of 0.413 μM and 0.657 μM, respectively.

Recently, Zhao et al. reported a set of EGFR PROTACs deriving from EGFR inhibitor.^90^ Treating HCC827 cell line with PROTAC 41 (Table 12) led to a significant loss of EGFR, and PROTAC 41 pronounced a potent and superior proliferation inhibition of HCC827 cell compared to AZD9291 and parent compound F. Furthermore, both EGFR^Del19^ and EGFR^L858R/T790M^ could be significantly induced to be degraded under treatment of PROTAC 41. This work would provide an alternative approach to the development of potentially effective EGFR degraders and provided a new clue to investigate PROTAC-induced protein degradation.

In 2021, Qu et al. described two degraders (PROTAC 42 and PROTAC 43, Table 12) by conjugating EGFR inhibitor canertinib and CRBN ligand pomalidomide.^91^ The reported degraders displayed potent and selective antitumour activities in EGFR-TKI-resistant lung cancer cells. They could selectively degrade EGFR^L858R+T790M^-resistant proteins in H1975 cells at the concentration of 30–50 nM and EGFR^Ex19del^ proteins in PC9 cells. In addition, these degraders showed better inhibition of EGFR phosphorylation in H1975 cells and PC9Brca1 cells compared to Canertinib. This finding suggested a promising approach to target EGFR to overcome clinical resistance.

PROTAC 44 (Table 12) with brigatinib as the warhead was illustrated by Ren et al. as an EGFR degrader in 2021.^92^ PROTAC 44 at approximately 100 nM was able to efficiently degrade both mutant EGFR^L858R + T790M^ and ALK fusion proteins (the two most important targets in non-small-cell lung cancer). In EGFR-expressing H1975 and ALK(G1202R) overexpressing 293 T-cell lines, PROTAC 44 exhibited better cell proliferation inhibition than brigatinib, with IC_50_ values of 42 and 21 nM in these two cell types, respectively. Furthermore, PROTAC 44 was orally bioavailable and well tolerated *in vivo*. PROTAC 44 was an enlightening degrader for them to tap into the fascination of protein degradation.

Hypoxia is a hallmark of many tumours and it leads to overexpression of various proteins such as EGFR. Many antitumour drugs have been designed to target hypoxia. In 2021, Cheng et al. reported the identification of a hypoxia-activated PROTAC (PROTAC 45, Table 12) by introducing a hypoxia-activated leaving group (1-methyl-2-nitro-1*H*-imidazol-5-yl)methyl or 4-nitrobenzyl into the structure of EGFR^Del19^-based PROTAC.^93^ PROTAC 45 exhibited stronger degradation activity against EGFR^Del19^ in HCC4006 cells under hypoxia than in normoxia. This was the first example of using tumour hypoxia to identify PROTACs that acted selectively on tumours, providing a new approach for PROTACs development.

#### 2.5.2. VHL-based PROTACs

In 2018, Burslem et al. reported a potent EGFR degrader, PROTAC 46 (Table 13), which consisted of the EGFR kinase inhibitor lapatinib and a VHL ligand.^22^ PROTAC 46 could degrade target protein with a DC_50_ = 39.2 nM and a D_max_ = 97.6% in the OVCAR8 cell line and revealed better antiproliferative effects in comparison to EGFR inhibitor. PROTAC 46 had potent antiproliferative efficacy in SKBr3 cells with IC_50_ = 102 nM. Importantly, PROTAC 46 also induced the degradation of exon-20 insertion mutant form of EGFR in the HeLa cell line. Gefitinib was used to replace lapatinib to develop PROTAC 47 (Table 13), which degraded exon-19 deletion EGFR (DC_50_ = 11.7 nM and D_max_ = 98.9%) in the HCC827 cell line and the L858R activating point mutation (DC_50_ = 22.3 nM and D_max_ = 96.6%) in the H3255 cell line. When afatinib was employed to develop PROTAC 48 (Table 13), it could degrade gefitinib-resistant mutant EGFR^L858R/T790M^ with DC_50_ = 215.8 nM and D_max_ = 79.1% in the H1975 cell line.

**Table 13.** Representative VHL-based PROTACs targeting EGFR.

Compounds	Target protein	Structure	Ref.
PROTAC 46	EGFR	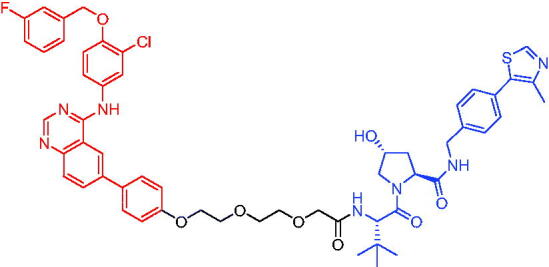	^22^
PROTAC 47	EGFR	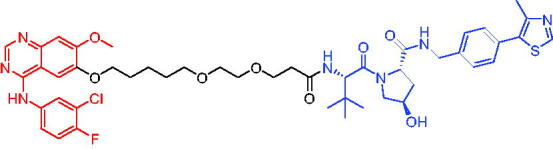	^22^
PROTAC 48	EGFR	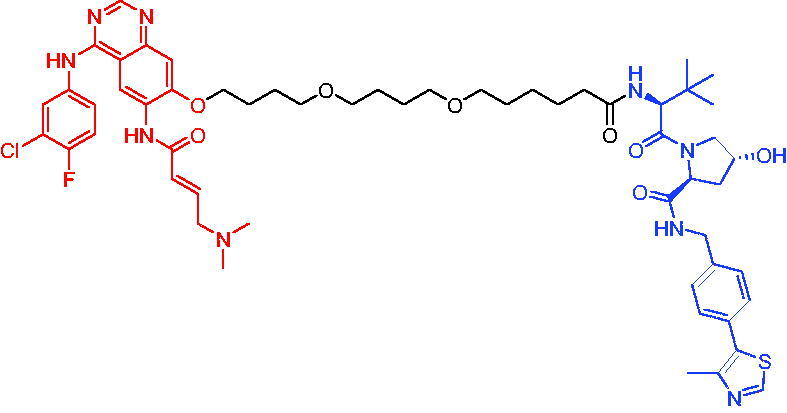	^22^
PROTAC 49	EGFR	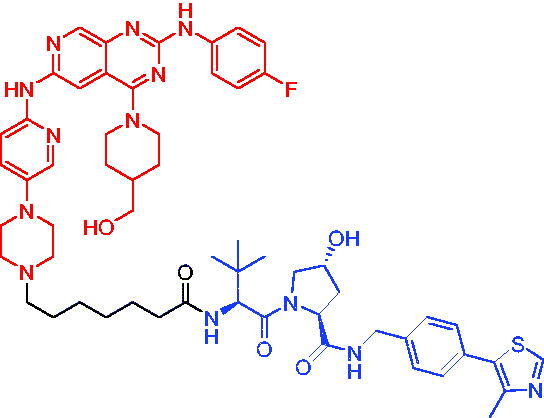	^87^
PROTAC 50	EGFR	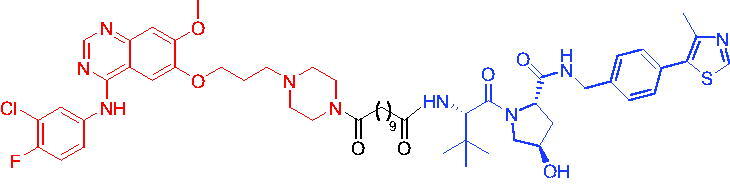	^88^
PROTAC 51	EGFR	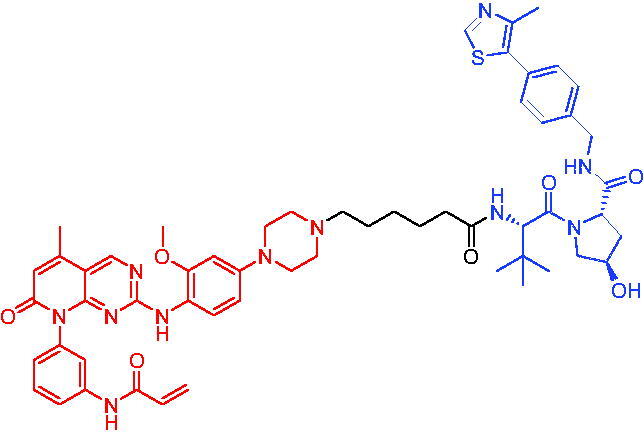	^94^
PROTAC 52	EGFR	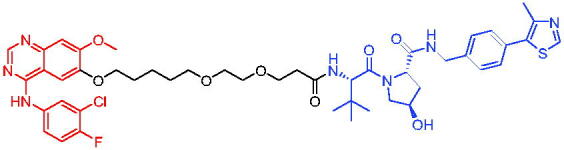	^95^
PROTAC 53	EGFR	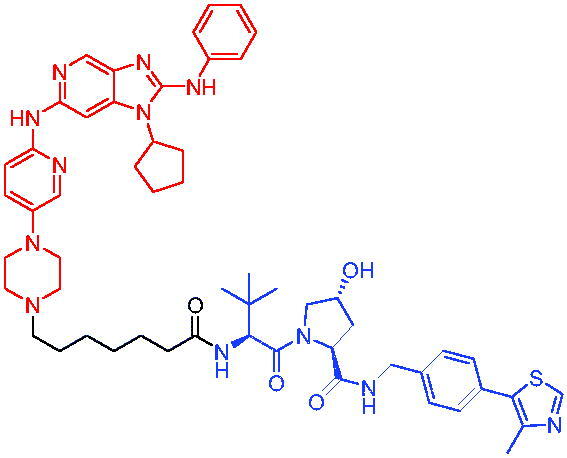	^90^
PROTAC 54	EGFR	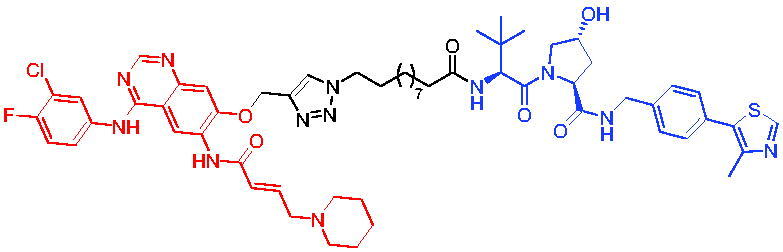	^96^

In 2020, Zhang et al. developed a novel EGFR degrader, PROTAC 49 (Table 13), through connection of a fourth-generation EGFR inhibitor (pyrido[3,4-*d*] pyrimidine) and a VHL E3 ligase ligand.^87^ PROTAC 49 induced efficient degradation of EGFR with a DC_50_ value of 34.8 nM in HCC827 cells. It also could significantly induce the apoptosis of HCC827 cells and arrest the cells in the G1 phase.

In the same year, Cheng et al. published another EGFR degrader PROTAC 50 (Table 13) based on gefitinib.^88^ PROTAC 50 showed better protein selectivity and potent protein degradation. In addition, PROTAC 50 was bioavailable in mouse pharmacokinetic studies, and was the first EGFR PROTAC suitable for *in vivo* efficacy studies. In conclusion, this study provided a set of well-characterised chemical tools to the research community.

Zhang et al. designed and developed a series of selective EGFR^L858R/T790M^ mutant degraders by conjugating pyrido [2, 3-*d*] pyrimidin-7-one selective EGFR^L858R/T790M^ inhibitor XTF-262 with an E3 ubiquitin ligase.^94^ In this work, they found that PROTAC 51 (Table 13) effectively and selectively reduced EGFR^L858R/T790M^ with a DC_50_ value of 5.9 nM, while did not show an obvious effect on the wild-type protein. PROTAC 51 could be used as an initial lead molecule for the development of new therapies based on EGFR^L858R/T790M^ PROTACs. Further pharmacokinetically oriented structural optimisation of PROTAC 51 was currently being performed by the authors and the results would be disclosed in due course.

EGFR overexpression and activating mutations in NSCLC H3255 cells can promote NSCLC resistance to immunotherapy by upregulating inhibitory immune checkpoints, such as programmed death receptor ligand 1 (PD-L1) and indoleamine-2,3-dioxygenase-1 (IDO1). Thus, selective inhibition of EGFR is also expected to modulate the immune microenvironment to advance NSCLC immunotherapy. Wang et al. reported a new multifunctional EGFR degrader by tethering the selective EGFR inhibitor gefitinib with a VHL ligand.^95^ PROTAC 52 (Table 13) reduced EGFR^L858R^ to investigate its potential in dually inhibiting PD-L1 and IDO1 to potentiate the antitumour immunity in NSCLC. PROTAC 52 significantly reduced the protein levels of PD-L1 and IDO1 in NSCLC H3255 cells and tumours compared to gefitinib. PROTAC 52 could have enhanced potency and specificity. In addition, PROTAC 52 significantly inhibited the growth of H3255 tumours and enhanced the antitumour immune response in H3255 tumours. Overall, the authors have demonstrated the potential of EGFR^L858/R^ PROTACs in enhancing the antitumour immune response in NSCLC. These findings provided a basis for future treatment of NSCLC with EGFR PROTAC alone or in combination with ICIs.

Recently, Zhao et al. developed a set of EGFR PROTACs based on a reversible EGFR-TKI with purine scaffold.^90^ PROTAC 53 (Table 13) induced remarkable both EGFR^Del19^ and EGFR^L858R/T790M^ degradation with DC_50_ values of 0.51 and 126.2 nM, respectively. Furthermore, PROTAC 53 showed potent antiproliferative activity against HCC827 and H1975 cell lines with IC_50_ values of 0.83 and 203.01 nM, respectively. Moreover, PROTAC 53 significantly induced apoptosis, blocked the cell cycle, and inhibited cell colony formation. The authors found that ubiquitination was indispensable in the degradation process and found that degradation was associated with autophagy. Their work would provide new ideas for the development of potentially effective EGFR degraders and provide new clues for the study of PROTAC-induced protein degradation.

In 2022, Shi et al. reported the discovery of dacomitinib-based EGFR PROTACs.^96^ PROTAC 54 (Table 12) could effectively induce degradation of EGFR^Del19^ with DC_50_ value of 3.57 nM in HCC-827 cells, but not to other EGFR mutant, wild-type EGFR protein and the same family receptors (HER2 and HER4). Noteworthily, PROTAC 54 was the first EGFR PROTAC to evaluate antitumour effect *in vivo*, and exhibited excellent antitumour efficacy (TGI = 90%) at a dose of 30 mg/kg without causing observable toxic effects. The preliminary mechanism study demonstrated that PROTAC 54 could efficiently degrade EGFR protein through ubiquitin proteasome pathway and inhibit phosphorylation of downstream pathways *in vitro* and *in vivo*, which indicated that PROTAC 54 exerted antitumour effect by degradation of EGFR protein in tumour tissue.

#### 2.5.3. IAP-based PROTACs

In 2020, a series of IAP-based EGFR^L858R/T790M^ mutant PROTACs were developed based on pyrido [2,3-d]pyrimidin-7-one selective EGFR^L858R/T790M^ inhibitor XTF-262.^95^ In contrast to reported PROTACs, IAP-based PROTAC (PROTAC 55, Table 14) was unable to degrade EGFR^L858R/T790M^ protein, which was not overly described by the authors.

**Table 14.** Representative PROTAC targeting EGFR.

Compound	Target protein	Structure	Ref.
PROTAC 55	EGFR	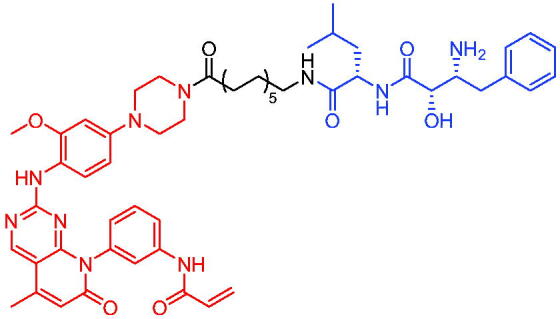	^95^

### 2.6. Targeting EGFR/PARP

Drug resistance in advanced cancers is mediated by different factors, such as overexpression of EGFR and DNA repair enzymes. Poly(ADP-ribose) polymerase (PARP) is a key protein in the known base excision repair (BER) and cellular signalling pathways.^97^ Inhibition of EGFR leads to downregulation of key players in BER and sensitises cells to alkylating drugs and ionising radiation. Like EGFR inhibitors, receptor tyrosine kinase inhibitors (TKIs) show promising therapeutic effects and are widely used in clinical practice. The emergence of drug resistance, such as that caused by T790M mutations, has greatly reduced its efficacy. EGFR-mutated cancer cells have been shown to be sensitive to olaparib both *in vivo* and *in vitro*. Therefore, inhibition of EGFR and PARP may have a synergistic effect.

#### 2.6.1. VHL-based PROTACs

In 2021, Zheng et al. reported their work on the development of dual EGFR and PARP degraders by merging EGFR inhibitor and PARP inhibitor with the E3 ligase ligand in one novel star-shaped molecule.^98^ PROTAC 56 (Table 15) degraded EGFR and PARP simultaneously in a dose-dependent manner in H1299 cells. At a concentration of 0.47 μM, PROTAC 56 degraded approximately 50% PARP, and it degraded higher levels of PARP as the concentration was increased. For EGFR, PROTAC 56 also degraded higher levels of degradation when PROTAC 56′ concentration was gradually increased. Moreover, PROTAC 56 significantly induced degradation of EGFR and PARP at 15 μM. This was the first successful example of dual PROTACs.

**Table 15.** Representative VHL-based PROTAC targeting EGFR/PARP.

Compound	Target protein	Structure	Ref.
PROTAC 56	EGFR/ PARP	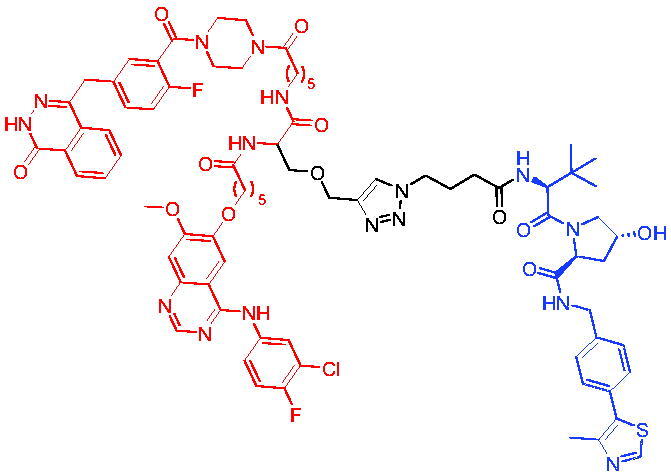	^98^

### 2.7. Targeting ER

Breast cancer is one of the most common malignancies in women. The vast majority of newly diagnosed breast cancer cases occur in ER + breast cancers. As members of the nuclear receptor family, the oestrogen receptors ERα and ERβ are transcription factors that regulate gene expression and mediate the biological effects of oestrogen. ERα is thought to be the primary mediator of oestrogen signalling in the female reproductive tract and mammary gland. Therefore, ERα has been pursued as a promising therapeutic target in cancer treatment. The current therapeutic agent is fulvestrant, which acts by selectively degrading oestrogen receptors in ER + metastatic breast cancer. However, after six months of treatment with fulvestrant, the therapeutic effect is greatly reduced. Therefore, new therapeutic agents are urgently needed to target oestrogen receptors.^99–101^

#### 2.7.1. VHL-based PROTACs

In 2019, Hu et al. reported the first VHL-based PROTAC targeting ER, based on fulvestrant and a VHL E3 ubiquitin ligase.^102^ PROTAC 57 (Table 16) showed better protein selectivity and potent protein degradation. Its DC_50_ were 0.17 and 0.43 nM and D_max_ was 95% in MCF-7 and T47D cells at 4 h, respectively. PROTAC 57 achieved more complete degradation than the only approved fulvestrant. Consistently, PROTAC 57 achieved more complete cell growth inhibition than fulvestrant in MCF-7 cells. They concluded that further optimisation of ER PROTACs might lead to a novel and effective class of therapeutic agents for the treatment of advanced and metastatic ER + breast cancer.

**Table 16.** Representative VHL-based PROTACs targeting ER.

Compounds	Target protein	Structure	Ref.
PROTAC 57	ER	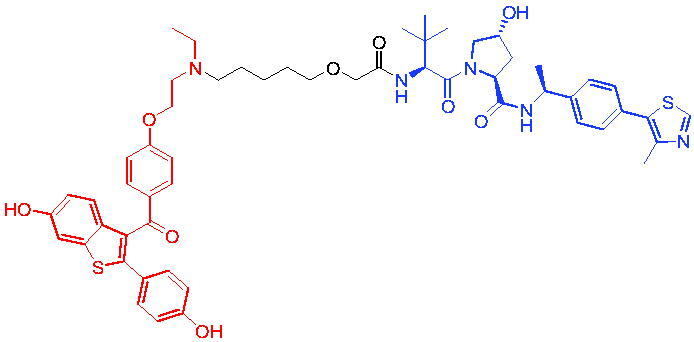	^102^
PROTAC 58	ER	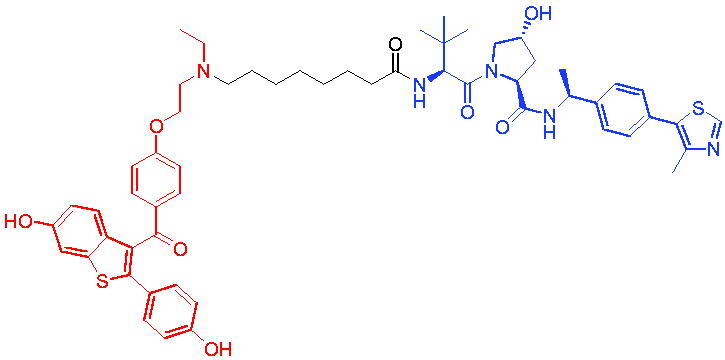	^103^
PROTAC 59	ER	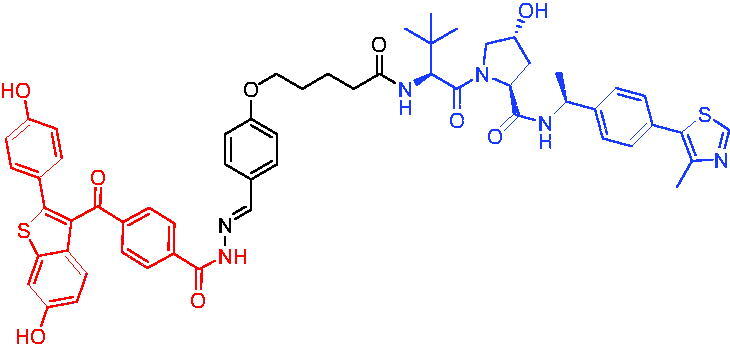	^104^
PROTAC 60	ER	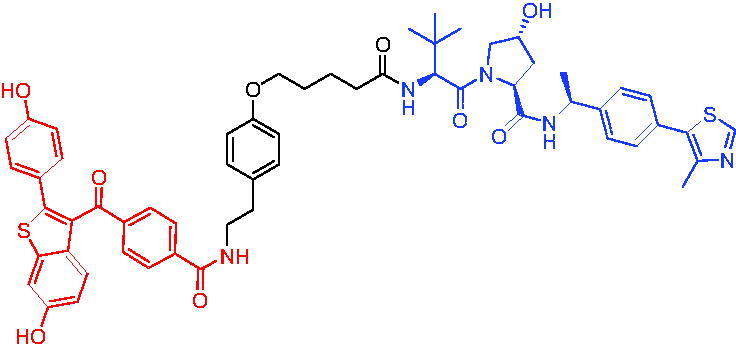	^104^
PROTAC 61	ER	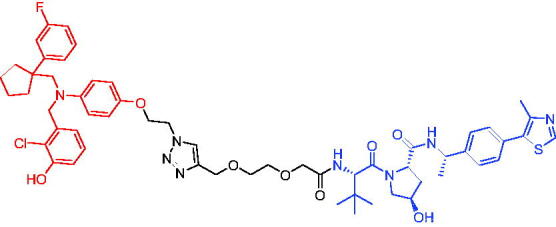	^105^
PROTAC 62	ER	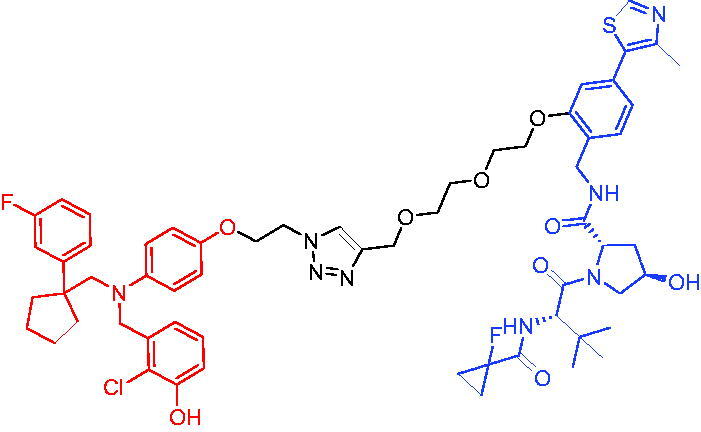	^105^

In 2020, Gonzalez et al. reported developing potent PROTACs tools based on a selective ER modulator raloxifene for selective degradation of ER protein.^103^ In this study, representative PROTAC 58 (Table 16) was the most potent degrader, which could significantly reduce the ERα protein level in parental MCF-7 and MCF-7 cells harbouring the CRISPR/cas9 knock-in LBD mutations. The expression of a critical ER-regulated gene, GREB1, was found to be significantly downregulated in ER + cell lines upon exposure to PROTAC 58 in a manner comparable to fulvestrant. As expected, evidence of possible resistance to PROTACs was observed in mutant cells and was shown in western blot and proliferation assays. Thus, the PROTACs strategy had become a highly desirable method for the modulation of ER levels.

Efficient PROTACs were found to require optimisation of many parameters, especially the type and length of linkers. In 2020, Roberts et al. reported their development of PROTACs targeting ER.^104^ In the first stage, nearly 100 PROTACs molecules were synthesised by simply mixing ER ligands containing a hydrazide functional group at different positions with pre-assembled VHL ligands bearing different types and lengths of linkers with a terminal aldehyde group in a 1:1 ratio. They found PROTAC 59 (Table 16) to be the most efficient ER degrader in both ER + cell lines (DC_50_ = 10 nM, D_max_ = 95%). The second stage involved the conversion to more stable amide linkers to produce more drug-like molecules. The optimally obtained PROTAC 60 (Table 16) showed comparable bioactivity (DC_50_ = 1.1 nM, D_max_ = 98%) and induced effective anti-diffusion in MCF-7 (IC_50_ = 13.2 nM, I_max_ = 69%). This proof-of-concept study demonstrated that a two-stage strategy could greatly facilitate the development of ER PROTACs without the cumbersome process of making a large number of PROTACs one by one.

In 2021, by using the DNA-encoded chemical library platform, Disch et al. identified some novel ERα binding agents that were efficiently integrated into VHL-involved PROTACs, exhibiting nanomolar ERα DC_50_ values in ER + cells, while showing no effect in ER- cells.^105^ The representative compounds PROTAC 61 and PROTAC 62 (Table 16) showed no off-target effects in normal immortalised mammary cells. In addition, PROTAC 61 and PROTAC 62 exhibited properties suitable for *in vivo* application and efficacy in ERα-dependent xenograft models. The discovery of these compounds could contribute to the development of novel ERα-based PROTACs for breast cancer.

#### 2.7.2. IAP-based PROTACs

In 2011, Itoh et al. published the first IAP-based degrader, PROTAC 63 (Table 17), by tethering the ER inhibitor oestrone to the IAP ligand bestatin.^106^ PROTAC 63 induced remarkable ERα degradation at 1 μM in human breast cancer cell MCF-7. Therefore, the development of novel protein degradation agents targeting the ER protein has become an excellent strategy.

**Table 17.** Representative IAP-based PROTACs targeting ER.

Compounds	Target protein	Structure	Ref.
PROTAC 63	ER	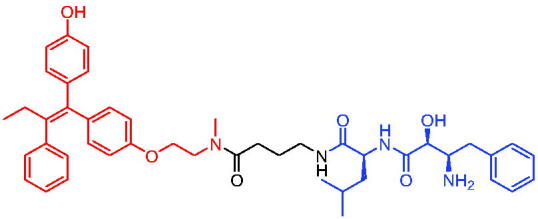	^106^
PROTAC 64	ER	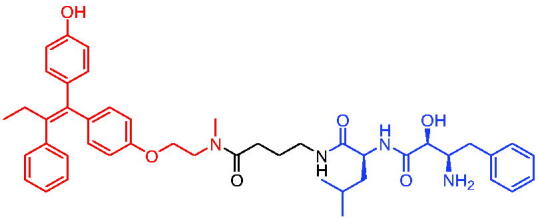	^107^
PROTAC 65	ER	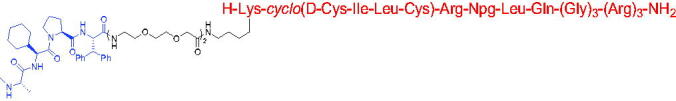	^108^
PROTAC 66	ER	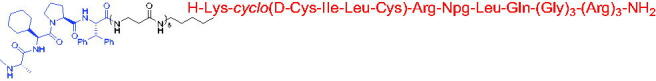	^108^
PROTAC 67	ER	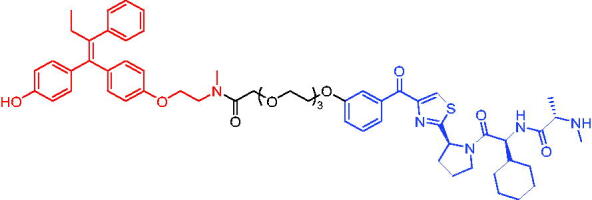	^109^
PROTAC 68	ER	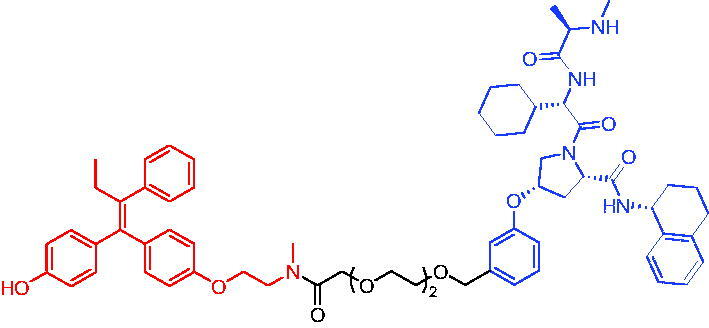	^110^
PROTAC 69	ER	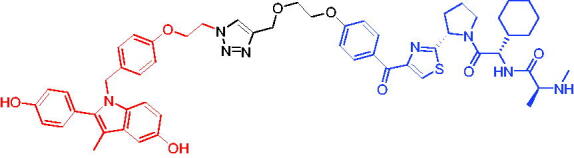	^105^
PROTAC 70	ER	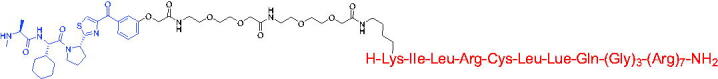	^111^
PROTAC 71	ER	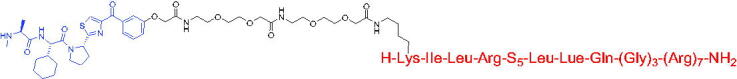	^111^

In 2012, Demizu et al. also reported some chemical ERα degraders, which contained 4-hydroxytamoxifen (4-OHT) and IAP ligand bestatin.^107^ PROTAC 64 (Table 17) potently degraded ERα at low concentration of 10 μM. PROTAC 64 was able to induce the production of reactive oxygen species in MCF-7 cells, which then led to cell death. In addition, the downregulation of ERa by PROTAC 64 was also observed in breast cancer cells T47D.

PERM3 is a peptide analogue of steroid receptor activator 1 (SRC-1) that reacts with the ER surface. R7 is a fragment of hepta-arginine that improves the permeability of PERM3. In 2016, Demizu et al. identified PROTAC 65 and PROTAC 66 (Table 17) by associating PERM3-R7 with MV-1.^108^ PROTAC 65 reduced ERα and cIAP1 levels in a concentration-dependent manner, but toxic effects began to appear at concentrations above 6 μM in MCF-7 cells. PROTAC 66 degraded ERα slightly less than PROTAC 65, but toxic effects appeared at concentrations of 20 μM.

The new degraders of ERα were developed by Ohoka et al. in 2017. Based on 4-OHT, ERα PROTACs were constructed that induced the degradation of ERα in the presence of different IAP-binding compounds (bestatin, MV1, and LCL161).^109^ After evaluation, the LCL161-derived PROTAC (PROTAC 67, Table 17) showed obvious degradation of ERα. Unlike IAP-based PROTACs described above, PROTAC 67 recruited XIAP rather than cIAP1 to ubiquitinate ERα for degradation. PROTAC 67 started to show degradation activity at 3 nM, with the best effect occurring at a concentration of 100 nM. In the MCF-7 tumour xenograft mouse model, PROTAC 67 significantly inhibited tumour growth without obvious toxic side effects.

In 2018, Ohoka et al. continued to describe some potent IAP-based PROTACs using new IAP inhibitors.^110^ Compared to PROTAC 67, the representative compound PROTAC 68 (Table 17) was more effective in inducing ERα degradation and apoptosis of MCF-7 in breast cancer cells. In addition, its ability to degrade ERα was superior to PROTAC 67 in MCF-7 xenograft mouse model.

In 2021, utilising DECL platform, Disch et al. described many IAP-based PROTACs targeting ERα.^105^ The representative compound PROTAC 69 (Table 17) was less efficient in degrading ERα proteins compared to the reported IAP-based PROTACs, which had not been studied much by the authors.

In 2021, Yokoo et al. successfully developed the stapled peptide stPERML-R7, which was based on the ERα-binding peptide PERML and consisted of natural amino acids.^111^ They developed a peptide-based degrader targeting ERα (PROTAC 70, Table 17), by conjugating stPERML-R7 with LCL-161. The chimeric peptide PROTAC 70 consistently degraded ERα and repressed ERα-mediated transcription more effectively than the unpinned chimaera LCL-PERML-R7 (PROTAC 71, Table 17). These results suggested that a stapled structure was effective in maintaining the intracellular activity of peptide-based PROTACs.

### 2.8. Targeting ER/GPER

GPER is a Gs-coupled heptahelical transmembrane receptor located at the plasma membrane and intracellular membrane that promotes rapid progenomic actions including activation of adenylate cyclases and transactivation of EGFRs. Stimulation of GPER facilitates the activation of signalling effectors downstream of EGFRs and is involved in cell proliferation, survival, invasion, and resistance to endocrine therapy. Its presence is associated with tumour progression, survival of breast cancer stem cells, and tamoxifen resistance. Thus, GPER broadens our ER-centric view of oestrogen responsiveness and undermines the binary criteria guiding the rational allocation of adjuvant therapy for breast cancer.^112^^,^^113^

#### 2.8.1. VHL-based PROTACs

In 2021, Lu et al. first reported two novel VHL-based degraders for knockdown of ERα, ERβ, and GPER.^114^ PROTAC 72 and PROTAC 73 formed high-affinity interactions with GPER and ER with binding dissociation constants of 30 nM and 10–20 nM, respectively. PROTAC 72 and PROTAC 73 (Table 18) effectively degraded plasma membrane and intracellular GPER and nuclear ER. Target specificity was further demonstrated in human MCF-7 cells, where both drugs effectively degraded ERα, ERβ, and GPER while ignoring the progesterone receptor (PR). In addition, PROTAC 72 and PROTAC 73 induced cytotoxicity and G2/M in MCF-7 breast cancer and human SKBR3 (ERα-ERβ-GPER+) breast cancer cells cell cycle arrest. These results suggested that it was possible to develop a number of receptor-based anti-oestrogen therapeutics for breast cancer that targeted both plasma membrane and intracellular oestrogen receptors.

**Table 18.** Representative VHL-based PROTACs targeting ER/GPER.

Compounds	Target protein	Structure	Ref.
PROTAC 72	ER/ GPER	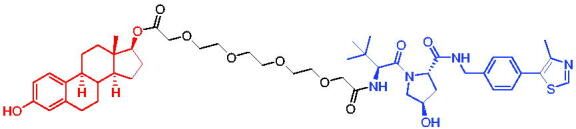	^114^
PROTAC 73	ER/ GPER	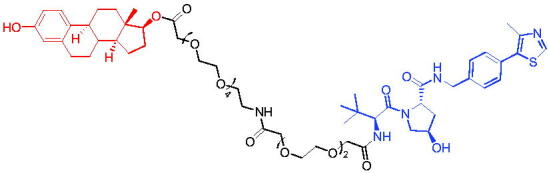	^114^

### 2.9. Targeting STAT3

STAT3 is one of the key members of the STAT family, which contains seven members including STAT1, STAT2, STAT3, STAT4, STAT5A, STAT5B, and STAT6. As a transcription factor, STAT3 plays a critical role in tumourigenesis by regulating genes related to cell survival, proliferation, invasion, and metastasis. STAT3 has emerged as a particularly attractive target for potential cancer therapy.^115^

#### 2.9.1. CRBN-based PROTACs

In 2019, Bai et al. designed and synthesised a series of potential PROTACs based on CRBN and STAT3 inhibitor SI-109 for the degradation of STAT3.^20^ PROTAC 74 (Table 19) could degrade >90% STAT3 in AML cells within 4 h and >50% STAT3 in ALCL cells. PROTAC 74 showed excellent selectivity compared to STAT3 inhibitors, as other members of the STAT family cannot be degraded or bound. PROTAC 74 potently degraded STAT3 xenograft tumours and achieved complete and durable tumour regression in mice. In addition, the authors found that PROTAC 74 caused profound depletion of STAT3 in mouse tissues, such as liver, spleen, heart, and kidney, but its safety profile appeared to be good.

**Table 19.** Representative CRBN-based PROTAC targeting STAT3.

Compound	Target protein	Structure	Ref.
PROTAC 74	STAT3	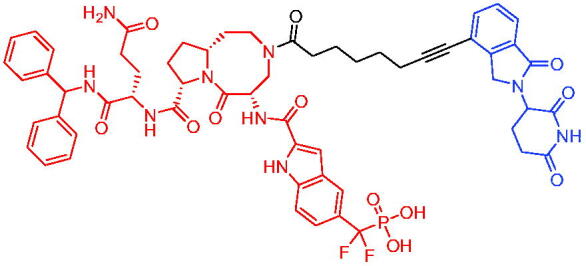	^20^

### 2.10. Targeting TRK

The tropomyosin receptor family kinases (TRK) include three important members, namely TRKA, TRKB, and TRKC, which are encoded by the NTRK1, NTRK2, and NTRK3 genes, respectively. Aberrant activation of the TRK pathway has been observed in different types of human cancers, with chromosomal translocations of the NTRK genes being the most studied with interest. Therefore, targeting TRK fusion proteins in human cancers holds great therapeutic promise.^116^^,^^117^

#### 2.10.1. CRBN-based PROTACs

In 2020, Chen et al. developed PROTAC 75 and PROTAC 76 (Table 20) as two first-in-class TRK degraders.^118^ PROTAC 75 and PROTAC 76 were capable of inducing the tropomyosin 3 (TPM3)-TRKA fusion protein degradation in KM12 colorectal carcinoma cells and inhibiting downstream PLCγ1 signalling at sub-nanomolar concentrations. They also degraded human wild-type TRKA with similar potency. Moreover, PROTAC 75 and PROTAC 76 were able to selectively degrade endogenous TPM3-TRKA without degrading ectopically expressed ATP/GTP-binding protein-like 4 (AGBL4)-TRKB or ETS variant transcription factor 6 (ETV6)-TRKC fusion proteins in KM12 cells. PROTAC 75 and PROTAC 76-induced degradation of TPM3-TRKA protein was further confirmed to be mediated through the CRBN and ubiquitin-proteasome systems. In addition, they exhibited higher potency in inhibiting the growth of KM12 cells compared to TRK kinase inhibitors.

**Table 20.** Representative CRBN-based PROTACs targeting TRK.

Compounds	Target protein	Structure	Ref.
PROTAC 75	TRK	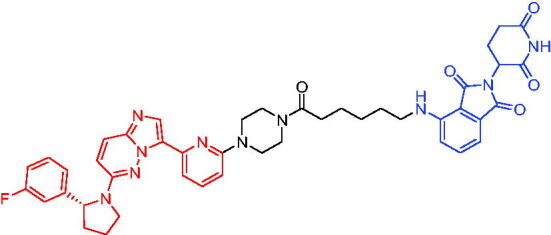	^118^
PROTAC 76	TRK	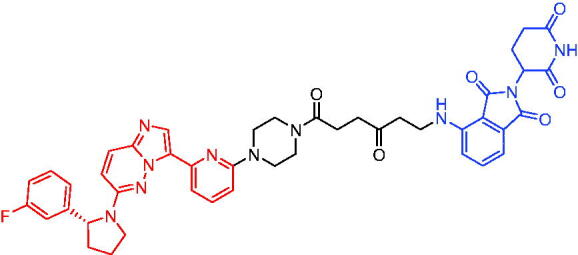	^118^

## 3. PROTACs for immune diseases

### 3.1. Targeting IRAK4

IRAK4 belongs to the IRAK kinase family (IRAK4, IRAK1, IRAK2, and IRAK-M). IRAK4 is a key molecule involved in the innate immune process, participating in transactivation pathways stimulated by Toll-like receptors (TLRs) and interleukin-1 (IL-1) family receptors. IRAK4 deficiency or loss of function has been reported to increase susceptibility to a number of pathogens, and kinase activation has been associated with various autoimmune diseases such as systemic lupus erythematosus, psoriasis, rheumatoid arthritis, and cancer.^119^^,^^120^

#### 3.1.1. CRBN-based PROTACs

In 2020, Zhang et al. published a series of novel CRBN-based PROTACs targeting IRAK4 by tethering a highly selective IRAK4 inhibitor and thalidomide.^121^ CRBN-based PROTACs showed moderate affinities to CRBN-DBB1, with Kd values ranging from 490 to 1080 nM. The representative degrader PROTAC 77 (Table 21) could efficiently degrade IRAK4 with a 90% D_max_ at 405 nM in HEK293T cells after 24-h treatment. PROTAC 77 was a useful tool to understand the scaffolding function of the IRAK4 protein, which was previously not possible with pharmacological perturbations.

**Table 21.** Representative CRBN-based PROTACs targeting IRAK4.

Compounds	Target protein	Structure	Ref.
PROTAC 77	IRAK4	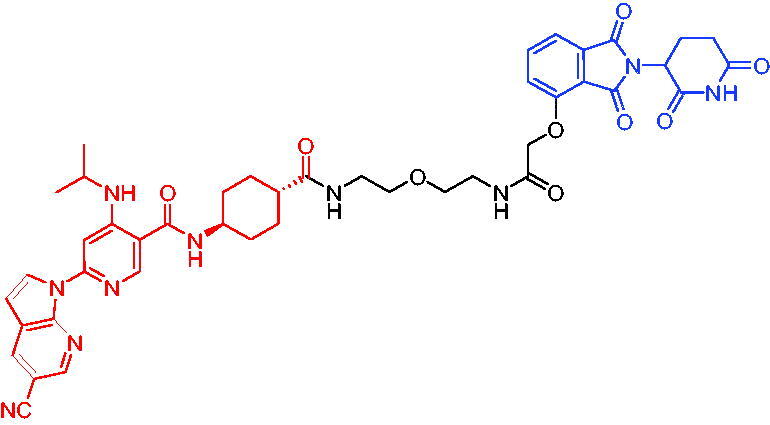	^121^
PROTAC 78	IRAK4	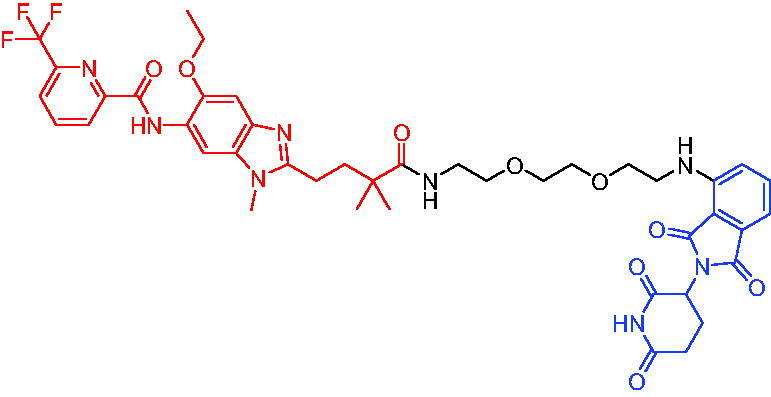	^122^

In 2021, Chen et al. varied the linkers of the bifunctional molecules to find IRAK4 PROTACs.^122^ The most potent degrader PROTAC 78 (Table 21), which was derived from pomalidomide and an IRAK4 inhibitor, showed specific and remarkable potency on IRAK4 degradation in OCILY10 and TMD8 cells. Moreover, PROTAC 78 efficiently blocked the IRAK4-NF-κB signalling pathway and displayed a substantial advantage in inhibiting the growth of cell lines expressing the MYD88 L265P mutant compared with the parent IRAK4 inhibitor.

#### 3.1.2. VHL-based PROTACs

In 2019, Nunes et al. reported a new IRAK4 degrader by conjugating PF-06650833 and the VHL ligand.^123^ The PROTAC-induced IRAK4 degradation was dependent on binding to VHL and was reversed upon blocking proteasome activity. In phenotypic assays measuring various inflammatory cytokines, PROTAC 79 (Table 22) and PF-06650833 had the same pharmacological profile. The authors believed that more work needed to be done to understand the biology of this target. Importantly, the discovery of novel strategies, such as PROTACs to target IRAK4, could not only support the understanding of IRAK4 biology but could also lead to the development of new therapeutic agents to treat inflammatory and neoplastic diseases.

**Table 22.** Representative VHL-based PROTAC targeting IRAK4.

Compound	Target protein	Structure	Ref.
PROTAC 79	IRAK4	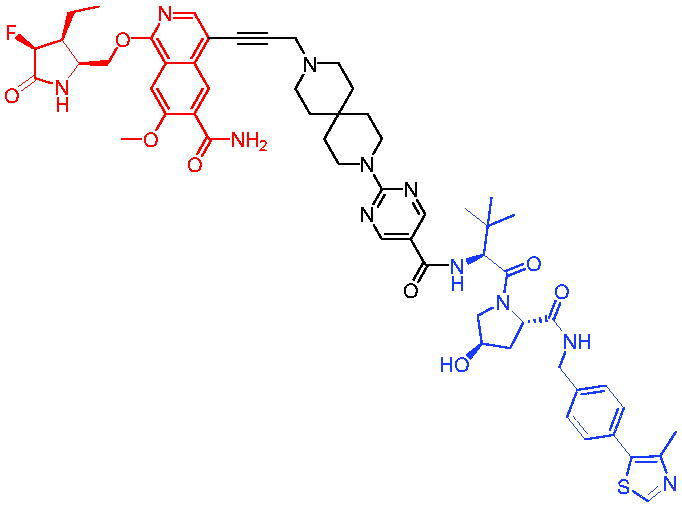	^123^

#### 3.1.3. IAP-based PROTACs

In 2019, Nunes et al. developed some IAP-based PROTACs targeting IRAK4 protein.^123^ The authors found that representative compounds PROTAC 80 and PROTAC 81 (Table 23) could not degrade IRAK4 protein. They suggested that there were many potential reasons for the inability of these compounds to degrade IRAK4. For example, the length of the linker might be wrong to promote efficient ternary complex formation. The orientation of the protein-IAP E3 ligase ternary complex might also fail to promote efficient transfer of ubiquitin to the lysine residues on the IRAK4 surface. Finally, even though these compounds might bind to the protein, this did not always translate into degradation.

**Table 23.** Representative IAP-based PROTACs targeting IRAK4.

Compounds	Target protein	Structure	Ref.
PROTAC 80	IRAK4	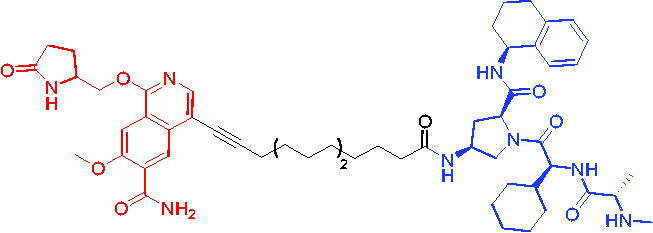	^123^
PROTAC 81	IRAK4	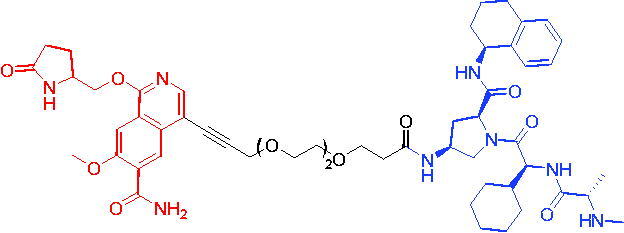	^123^

## 4. Conclusions and perspectives

During this past period, PROTACs have emerged as a novel strategy for disease treatment that employs UPS to induce selective degradation of target proteins by hijacking E3 ligases. PROTACs consist of three parts: the ligand of the target protein, the ligand of the E3 ligase, and the linker between them. These three components are crucial for the therapeutic efficacy of PROTACs. Compared with other drugs, PROTACs show many advantages. First, PROTACs can induce degradation of pathogenic proteins, which facilitates multiple rounds of target proteins degradation and may help to eliminate off-target effect. Second, PROTACs can also degrade proteins that cannot currently be treated by drugs, such as scaffolding proteins and transcription factors. Third, PROTACs can overcome drug resistance, which has been proved by PROTACs targeting AR, ER, BTK, etc. Recently, PROTACs have entered clinical studies as degraders of many target proteins (such as AR, BCL-XL, BRD9, BTK, EGFR, ER, IRAK4, STAT3, and TRK). Although PROTACs have many advantages in clinical applications, challenges including oral bioavailability, PK/PD/efficacy relationships, distribution, metabolism, and toxicity need to be addressed. First, most of the reported PROTACs have high molecular weights that do not qualify as potential therapeutic agents. Second, the mechanisms of PROTACs are not well studied and more practice needs to be done. Third, more than 600 E3 ligases have been reported to be identified in humans, but less than 1% of them have been successfully used due to the lack of small-molecule ligands. To date, the vast majority of reported PROTACs induce target protein degradation by recruiting E3 ligases CRBN, VHL, MDM2, and IAP, and there is an urgent need to develop PROTACs with more E3 ligase ligands. Fourth, linkers are also critical for the degradation activity of PROTACs, including membrane permeability and metabolic stability. Up to now, the principles guiding linker design, including length and composition, have not been rigorously mastered. Considerable work is needed to obtain optimal linkers. Although PROTACs have many challenges to address, they have the potential to be developed as therapeutic agents for many difficult-to-treat diseases. Excitingly, to date, at least six companies have brought PROTACs molecules into clinical trials, which has greatly encouraged researchers in the pharmaceutical and academic fields. We believe that these existential challenges will be successfully addressed in the future with continued efforts on PROTACs technology.
